# Resurrection of Wheat Cultivar PBW343 Using Marker-Assisted Gene Pyramiding for Rust Resistance

**DOI:** 10.3389/fpls.2021.570408

**Published:** 2021-02-11

**Authors:** Achla Sharma, Puja Srivastava, G. S. Mavi, Satinder Kaur, Jaspal Kaur, Ritu Bala, Tarvinder Pal Singh, V. S. Sohu, Parveen Chhuneja, Navtej S. Bains, G. P. Singh

**Affiliations:** ^1^Department of Plant Breeding & Genetics, Punjab Agricultural University, Ludhiana, India; ^2^School of Agricultural Biotechnology, Punjab Agricultural University, Ludhiana, India; ^3^Office of Director Seeds, Punjab Agricultural University, Ludhiana, India; ^4^Indian Institute of Wheat & Barley Research, Karnal, India

**Keywords:** wheat breeding, PBW343, leaf rust, stripe rust, gene pyramiding, marker assisted selection

## Abstract

Wheat variety PBW343, released in India in 1995, became the most widely grown cultivar in the country by the year 2000 owing to its wide adaptability and yield potential. It initially succumbed to leaf rust, and resistance genes *Lr24* and *Lr28* were transferred to PBW343. After an unbroken reign of about 10 years, the virulence against gene *Yr27* made PBW343 susceptible to stripe rust. Owing to its wide adaptability and yield potential, PBW343 became the prime target for marker-assisted introgression of stripe rust resistance genes. The leaf rust-resistant versions formed the base for pyramiding stripe rust resistance genes *Yr5, Yr10, Yr15, Yr17*, and *Yr70*, in different introgression programs. Advanced breeding lines with different gene combinations, PBW665, PBW683, PBW698, and PBW703 were tested in national trials but could not be released as varieties. The genes from alien segments, *Aegilops ventricosa* (*Lr37/Yr17/Sr38*) and *Aegilops umbellulata* (*Lr76/Yr70*), were later pyramided in PBW343. Modified marker-assisted backcross breeding was performed, and 81.57% of the genetic background was recovered in one of the selected derivative lines, PBW723. This line was evaluated in coordinated national trials and was released for cultivation under timely sown irrigated conditions in the North Western Plain Zone of India. PBW723 yields an average of 58.0 qtl/ha in Punjab with high potential yields. The genes incorporated are susceptible to stripe rust individually, but PBW723 with both genes showed enhanced resistance. Three years post-release, PBW723 occupies approximately 8–9% of the cultivated area in the Punjab state. A regular inflow of diverse resistant genes, their rapid mobilization to most productive backgrounds, and keeping a close eye on pathogen evolution is essential to protect the overall progress for productivity and resistance in wheat breeding, thus helping breeders to keep pace with pathogen evolution.

## Introduction

Bread Wheat (*Triticum aestivum*) is one of the most important cereals consumed worldwide and is a major crop of India, which forms an important part of the daily diet along with rice. Wheat yield has increased substantially in the past few years, and this appreciable yield increase may be attributable to the development of high yielding and rust-resistant varieties bred under the collaborative efforts of International and National institutions (Sendhil et al., 2019). According to future projections, India needs to lift its annual food production to 333 million tons by 2050 to feed the population (Pingali et al., 2019) from the current level of 296 million tons (3rd advance estimates, 2019–2020). India is the second largest producer of wheat worldwide (Sharma and Sendhil, 2016) with approximately 30 million hectares (14% of the global area) under wheat cultivation with the highest output of 106.21 million tons during 2019–2020 and a record average productivity of 3371 kg/ha (Sendhil et al., 2019). To meet these projections, the wheat breeding program at Punjab Agricultural University (PAU), Ludhiana, Punjab, India, has mainly focused on productivity enhancement and productivity protecting mechanisms. The emergence of new pests and diseases is a continuous threat to food sustainability, and the situation is further aggravated by the changing climate, which might trigger the emergence of new races of pathogens. Wheat rust diseases continually pose a threat to global wheat production (Khan et al., 2017). Stripe rust, caused by *Puccinia striiformis f. sp. tritici*, is an important disease of wheat in India and has caused severe epidemics in the past, leading to heavy economic losses (Kumar et al., 2020). The intensity of loss primarily depends upon the resistance level of the cultivars (Figueroa et al., 2018). Similarly, leaf rust or brown rust caused by the heteroecious basidiomycete *Puccinia triticina*, is another major rust disease and its epidemics cause significant yield losses (German et al., 2007; Figueroa et al., 2018). Rust diseases have been a challenge to plant breeding programs in India because of their ability to cause high yield losses if not controlled (Prashar et al., 2015). In India, the North Western Plain Zone (NWPZ) comprising the Indo Gangetic plains of India is the main wheat producing region. Punjab, a geographically small state in this region, is known as the food bowl of the country, which is testified by the fact that 40–60 percent of wheat is contributed to the national food reserves by the Punjab state alone. Wheat is the predominant grain crop in Punjab, which is grown on an area of around 35 million hectares and occupies about 90 percent of the total cropped area in the season. Besides being the major wheat producing state, Punjab also has been identified as a hot spot for stripe rust occurrence (Kaur et al., 2018). The development and deployment of cultivars with host genetic resistance are the most economical, effective, and environmentally friendly methods to reduce damage and loss caused by rust.

Wheat cultivar PBW343, an Attila sib (ND/VG9144//KAL/BB/3/YACO/4/VEE#5), was developed at CIMMYT, Mexico. It was released for general cultivation under timely sown irrigated conditions in the entire NWPZ, including Punjab, in 1995. PBW343 emerged as a mega cultivar on account of its adaptability to more comprehensive environments and hence, wider cultivation for more than a decade. PBW343 produced a higher number of grains per square meter, was photosynthetically more active, and kept its canopy cool on account of higher stomatal conductance (Joshi et al., 2007), which enabled it to spread further into heat stress environments and late sown conditions. Owing to its wider adaptation, which can also be credited to the presence of 1B/1R wheat-rye translocation reported to enhance adaptability (Rajaram et al., 1983), PBW343 performed exceptionally well in the North Eastern Plains Zone of India and was released in the year 2000 for cultivation in that zone. By the year 2002–2003, PBW343 occupied more than 90% of the wheat-growing area in Punjab and about 7 million hectares across the North West Plains Zone of India. The economic benefit of PBW343 to farmers in the NWPZ was estimated to be about 419 million USD, within the first 5 years of its cultivation (Anonymous, 2012).

A race of *P. striiformis*, with virulence for the gene *Yr9*, was first observed in East Africa in 1986 and subsequently migrated to North Africa and South Asia. Once it appeared in Yemen in 1991, it took just 4 years to reach the wheat fields of South Asia (Singh and Huerta-Espino, 2000). Most of the cultivars being grown at that time were susceptible to *Yr9* virulence, and consequently, considerable losses in wheat production occurred in almost all the major wheat-growing regions of North Africa, Central and Western Asia, and South Asia. However, PBW343 replaced the cultivars that had become susceptible to the race with *Yr9* virulence. By virtue of the stripe rust-resistant gene *Yr27*, derived from Selkirk, PBW343 withstood the spread of *Yr9* virulence, to which many other Veery derivatives succumbed (McIntosh et al., 2003; McDonald et al., 2004). Large-scale cultivation of PBW343 over approximately 10 million hectares facilitated the selection of virulence for *Yr27*, designated as ‘78S84’ (Prashar et al., 2007). Similarly, *Yr27* virulence emergence and its movement followed the path of the *Yr9* virulent races and affected the wheat production in India. Also, PBW343 had showed signs of the breakdown of leaf rust resistance in the field in the years 2003–2004 and 2004–2005, though seedling susceptibility to leaf rust pathotype 77-5 was detected about 4 years earlier (Chhuneja et al., 2005).

Constant and continued efforts were underway to create a rust-resistant variety for the protection of the yield in the region. Apart from improving PBW343, the wheat breeding program at PAU released several other cultivars, viz. DBW17 (2007), PBW 550 (2008), PBW621 (2011), and HD2967 (2011) yielding better results than PBW343. Each of these succumbed to stripe rust pathogen races within 3 years of their release. The current varietal spectrum represented a transition from PBW343 to this next set of varieties, but the evolution of the pathogen was so quick that it not only rendered PBW343 susceptible to stripe rust but also slowly increased the susceptibility of all the newly released varieties. The current study documents the revival of PBW343 and development of *Unnat* PBW343 using a gene stacking strategy for bringing together two pairs of alien rust-resistant genes, *Lr37/Yr17* and *Lr76/Yr70*, in this popular Indian variety.

## Materials and Methods

### Plant Material

PBW343 was used as the base genotype in this study for the incorporation of various leaf and stripe rust resistance genes. The leaf rust resistance genes *Lr37* (linked with *Yr17 and Sr38*), *Lr24*, and *Lr28* were available in the background of the Thatcher cultivar from the leaf rust differential set. A parallel set for the stripe rust genes *Yr5, Yr10*, and *Yr15* were available in background Avocet-based differentials. PBW343 was used as the female recipient parent in all the crosses. The gene *Yr70/Lr76* was identified, mapped, and tagged in-house at PAU and is available in WL711-*Ae. umbellulata* NILs (Chhuneja et al., 2008; Bansal et al., 2020). Later, PBW343 + *Lr24* + *Lr28*, PBW343 + *Lr37*/*Yr17/Sr38*, and PBW343 + *Lr76/Yr70* acted as donors to pyramid these genes. The checks (HD2967, DPW621-50, WH1105, and HD3086) used in the yield trails were the same high yielding varieties used in the national level trials.

### Crossing and Accelerated Development of Homozygous Lines

**FIGURE 1:**
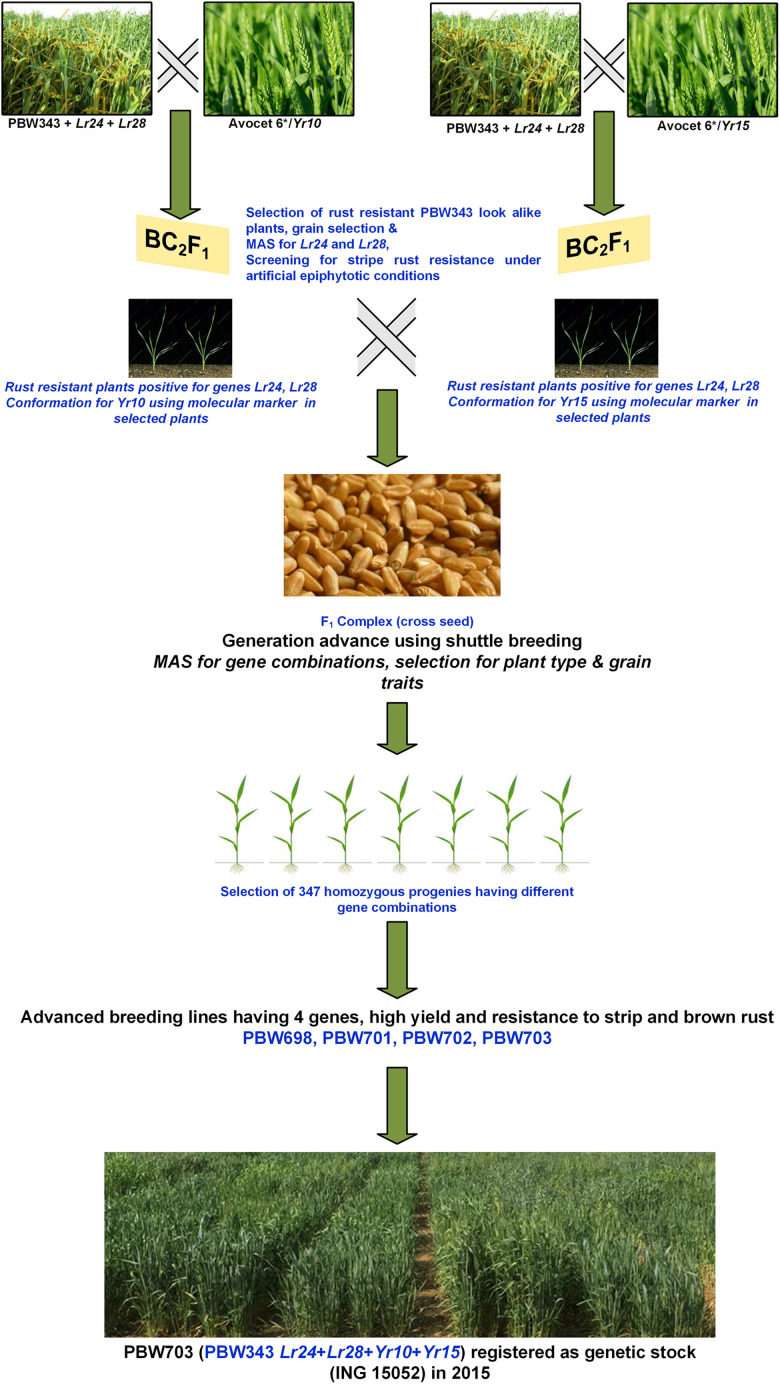
Schematic representation of the development of the four-gene pyramided line PBW703.

**FIGURE 2:**
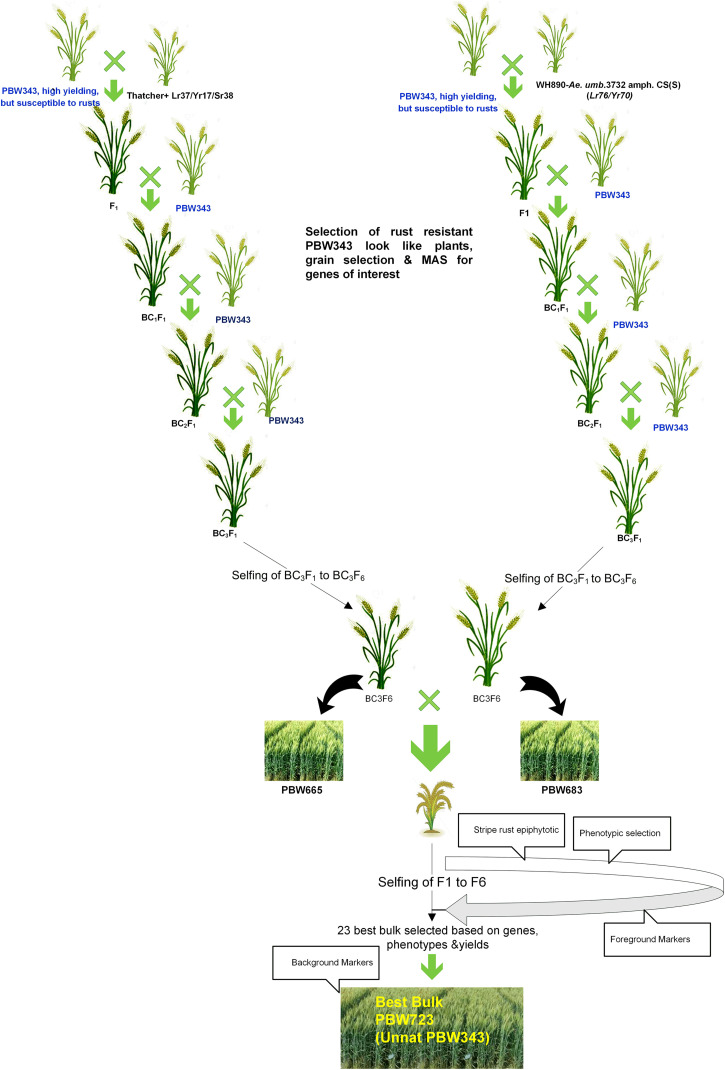
Schematic representation of the development of PBW723 alias *Unnat* PBW343.

Creating the *Unnat* (improved) PBW343 alias PBW723 from wheat variety PBW343 involved several attempts to develop a commercially viable variety. Single gene NILs for various leaf rust and stripe rust resistance genes were developed in the background of PBW343 by crossing and backcrossing respective donor lines for different resistance genes. Our first attempt marked the development of PBW343 near-isogenic lines for leaf rust resistance genes *Lr*24 and *Lr28* (Chhuneja et al., 2005) individually. The second attempt marked the pyramiding of both these genes resulting in the development of BW9250 (Chhuneja et al., 2011). The third attempt aimed at adding stripe rust genes *Yr10* and *Yr15* individually (PBW343 + *Lr24* + *Lr28* + *Yr10* and PBW343 + *Lr24* + *Lr28* + *Yr15*), while in the fourth attempt, these were pyramided to form a four-gene stack (PBW343 + *Lr24* + *Lr28* + *Yr10* + *Yr15*) line. The next attempt was in regard to adding the *Yr5* gene to PBW343. With the availability of more resistance genes, the sixth and seventh attempts gave the single gene NILs viz, PBW343 + *Lr37/Yr17, Lr57/Yr40*, and *Yr70/Lr76.* The eighth and final step was to pyramid these genes, which led to the development of PBW723 (*Unnat* PBW343). All the individual crosses and pyramided crosses were made at the ‘Wheat Experimental Area,’ PAU Ludhiana. In each case, the off-season facility in the Himalayas, at Keylong, Lahaul, and Spiti District, Himachal Pradesh (32°21′N latitude and 77°14′E longitude, 10,500 ft above mean sea level), India was used for generation advancement, which helped in attaining homozygosity in 3–3.5 years (6–7 seasons). The crossing scheme and development of PBW703 and PBW723 are given in Figures 1, 2. The crossing blocks and segregating populations in the field were maintained using the wheat agronomic practices package developed by PAU.

### DNA Extraction, PCR Analysis, and Resolution of the PCR Products

The molecular markers were applied for monitoring the genes of interest in the case of gene pyramiding. A phenotypic reaction under rust epiphytotic was used for selection while single gene introgressions and these progenies were later confirmed with linked markers for the presence of the gene(s). For the development of PBW723, the strategy given by Randhawa et al. (2009) was applied with some modifications. The details of the genes and molecular markers used for each gene are given in Supplementary Table 1.

The DNA of the segregants/lines was isolated from young leaves after rust screening by a modified CTAB method. PCR reactions for SSR/STS/SCA markers were performed in a total volume of 10 μl, containing 1 × PCR buffer, 200 μM of each dNTP, 20 ng of each primer, 1 U of Taq DNA polymerase, and 80 ng of genomic DNA. Amplified PCR products of these markers were resolved in 2–3% agarose gels, stained with ethidium bromide [The PCR reaction with Kompetitive allele-specific PCR (KASP)] markers were performed in a 96-well PCR plate in a volume of 4 μl as per the recommendations by LGC Genomics (2 μl of DNA, 1.944 μl of 2 × KASP mix, and 0.056 μl of primer mix;v4, LGC Genomics, Teddington, United Kingdom). Fluorescence of the plate was read on a Tecan Safire plate reader and the readings were imported on Kluster Caller software to check the cluster formation.

### Screening Against Leaf Rust and Stripe Rust

The screening of segregating progenies was done under artificial rust epidemics created by spraying the experimental material with a mixture of uredinospores of prevalent stripe and leaf rust pathotypes. The seeds were planted in a non-replicated augmented block design with paired rows of 1 m. The distance within the paired row was 0.22 m and between two paired rows was 0.3 m. The planting was done in the first fortnight of November each year. The check cultivars, PBW343, Agra local, and A-9-30-1 (for stripe rust), and C306 and Agra local (for brown rust), susceptible to a mixture of prevalent pathotypes with virulence for genes under consideration were planted as infector rows (at every 5th paired row) and in spreader rows (perpendicular to the 1 m rows) surrounding the plot for establishing sufficient inoculum and uniform disease development. To ensure uniform disease distribution, rust infected pots were placed in fields between the experimental materials, and the spores that appeared naturally in the spreader rows were used to inoculate the infector rows. Stripe rust epidemics were created by repeated spray inoculations with the uredinospores of *Puccinia striiformis.* Infected leaves of the susceptible host (PBW343/Agra local/A-9-30-1 which were pre-inoculated to multiply the pathogen) were taken and immersed in water to extract uredinospores. The inoculum was prepared by suspending rust uredinospores in 10 l of water using a few drops of Tween-20. Uredinospores were applied at a rate of 5.6 g/ha, which equates to 1000 spores per plant (Imtiaz et al., 2003). The spray inoculations were done in the evening with an ultralow volume sprayer on alternate days beginning from the end of December to the end of January until stripe rust appeared on the susceptible check/parent.

The response to rust was recorded at the reproductive stage using disease severity (DS) and infection response (IR) as the two measures according to the modified Cobb scale (Supplementary Table 2) given by Peterson et al. (1948). DS was measured as an estimation of the percentage coverage (0, 5, 10, 20, 40, 60, 80, and 100) of rust pustules (uredinia) seen on the flag leaf. IR was scored as a reaction of the host to rust infection and was categorized as 0 = immune; R = resistant, MR = moderately resistant; MS = moderately susceptible; and S = susceptible. Data were recorded three times at equal intervals (starting mid-January) when the flag leaves of the susceptible check cultivars showed a disease score of 80S (DS: 80; IR: S). Out of these three scores in a test line, the highest score toward susceptibility was used for the subsequent analysis. Once homozygous resistant lines were identified, screening against leaf rust and stripe rust was done both at the seedling stage and adult plant stage. The testing for stripe rust and leaf rust was done in separate nurseries maintained at geographical distances. Screening of the adult plant stage was done against a mixture of known races viz., 78S84, 49S119, and 110S119 for stripe rust and races 77-5 and 104-2 for brown rust (documented by Directorate of Wheat Research, Regional Station, Flowerdale, Shimla, Himachal Pradesh, India), and unknown races (collected from a farmer’s field, the details of races and their virulence formula are given in Supplementary Table 3). The Indian differential set used in the study for stripe rust is given in Supplementary Table 4. In addition, the *YR17*/6^∗^AOC (-32); AVOCET NIL for *Yr17;* and PBW683 [WH 890-*Ae*. *umb* 3732 amph/CS (S)//WL 711 NN/3/3^∗^PBW 343] for *Yr70* were included to check the reaction of prevalent pathotypes of P. striiformis on the genes used in the study.

Screening at the seedling stage was done under controlled conditions. First observations were recorded 3 weeks after inoculation when susceptibility reached a 20 percent severity. Final disease severity was recorded when a susceptible parent (PBW343) recorded the highest severity of 80-100S. Repeated observations on disease progression were recorded at regular intervals. From these observations AUDPC (area under disease progress curve) was calculated by using the formula suggested by Wilcoxson et al. (1975).

$$AUDPC=\sum_{i=1}^{k}1/2\left(Si+Si-1\right)\times d$$

where,

Si = disease severity at the end of time i

Si-1 = disease severity at the end of time i-1,

k = number of evaluations of disease and,

d = interval between two evaluations.

### Strategy Used for the Development of PBW665, PBW683, BWL3274, and BWL3275 Using Marker-Assisted Selection (MAS)

Crosses were made in the main crop season for each of these individually and *F*_1_’s were advanced at Keylong. The *F*_2_ single seed was space-planted and an artificial epiphytotic from a mixture of prevalent races was created for evaluating disease scores. A phenotypic reaction to a mixture of stripe rust was observed and resistant plants were identified. The elite plant types with resistance were selected and advanced at Keylong. Another cycle of rust screening was accomplished during the off-season, and since Keylong is in Himalayas, stripe rust naturally occurs. The *F*_3_ and *F*_5_ ‘plant to progeny’ rows were selected during the off-season while *F*_4_ and *F*_6_ were selected under field epiphytotic conditions based on agronomically desirable progeny, resistance, plant height, and grain traits in the main crop season. The foreground markers for genes effective against stripe rust and leaf rust were used to confirm the presence of gene(s).

### Strategy Used for the Development of PBW723 Using Marker-Assisted Selection

A marker-assisted backcross selection (MABS)-based gene introgression approach in wheat as described by Randhawa et al. (2009) was used with certain modifications. A large *F*_2_ (2780 plants) was planted under artificial stripe rust epiphytotic conditions for selection. Since, individually both genes *Yr70* and *Yr17* would show rust ranging from 20S to 40S, we considered completely resistant plants to indicate the presence of both the genes. Agronomically elite and rust free plants (190) were selected and foreground molecular markers were applied for the confirmation of genes under consideration. A total of 190 plants out of 2780 plants represented the PBW343-like rust resistant plants. The number of loci being incorporated in our study was five (from two alien segments), therefore only two foreground markers were required. The molecular genotyping for genes *Yr17/Lr37/Sr38* and *Yr70/Lr76* confirmed the presence of both the loci in 153 plants. Plants to progeny rows were sown from these and phenotypically PBW343-like plants were selected. Finally, 23 progenies were retained and evaluated for yield. The MABS approach (Randhawa et al., 2009) involves the intensive use of markers for foreground genes and background recovery. We used marker-assisted foreground selection for all the steps but as a modification, the marker-assisted background testing was conducted only on the best line. However, the phenotypic selection for resemblance to PBW343 was executed in segregating generations. Only the plants resembling the elite parent (PBW343) and those containing genes of interest (monitored via MAS) were retained during selections. For background selection, a total of 575 markers were used (Supplementary Tables 5–7), with many markers having copies. The A, B, and D marker loci were amplified, spanning all three genomes. A total of 197 amplifying fragments were used across seven chromosomes of the A genome, 215 for the B genome, and 163 markers to survey the D genome.

### Agronomic Evaluation and Yield Testing

The field experiments at PAU were conducted at the ‘Wheat Experimental Area,’ PAU Ludhiana, India (30° 54′ N latitude, 75°C 48′ E longitude, and 247 m above m s l). The soil of this region is of a mostly loamy sand type with a neutral pH (∼ 6–8). The climate of the location (Ludhiana), where the experiments regarding germplasm development and initial evaluation were conducted, is sub-tropical and semi-arid with cold winters from November to January, mild climate during February and March, and very hot with low moisture conditions during summer from April to June, followed by hot and humid conditions from July to September. The daily minimum temperature ranges from 0 to 4°C in January and the maximum temperature during May ranges from 39 to 45°C. The wheat growing season is from the last week of October to April, with crop being sown from the last week of October to the third week of November for timely sown conditions and as late as the first fortnight of January for late/very late sown conditions. The crop is harvested in the middle to end of April. All the experimental material to be evaluated under timely sown irrigated conditions was planted during the first fortnight of November, while for late sown crop, sowing was completed from December 5–15. The field was plowed, harrowed, and planked to obtain a fine tilth. The crop was raised using a standard package of practices as developed by PAU. A total of 110 kg of urea and 55 Kg of di-ammonium phosphate are recommended for wheat crop, while no muriate of Potash is added as soils are naturally rich in potassium in Punjab. Half of the nitrogen and full phosphate was applied at the time of sowing while the other half was applied in two equal splits, first at the crown root initiation stage and the remaining at the maximum tillering stage, coinciding with the first and second irrigations, respectively. Insecticide ‘thiamethoxam (Actara@50gm/ha)’ was applied to control aphids. Initially, pre emergence weedicide ‘pendimethalin (Stomp@3.75L/ha)’ was applied after sowing and later, the recommended herbicides were used for specific weed control when and where required. However, weeds were controlled manually in disease evaluation experiments to avoid any chemical effect on rust disease development.

The rust-resistant homozygous progenies positive for the genes of interest were bulked for preliminary yield testing along with standards checks. The plot size was 4.5 m × 6 rows, with three replications in each case. The markers and generation advancement through shuttling had reduced the time for first testing for each product. After a preliminary evaluation, the promising lines were tested at multiple locations (three sites: Ludhiana, Bathinda, and Gurdaspur) in Punjab with simultaneous testing in a plant pathology screening nursery at the national level (data not given). The resistant lines that gave superior yields were entered into the national coordinated program for testing. The lines were tested under coordinated testing in NIVT (National Initial Varietal Trial, 1 year, >40 locations, 6 m × 6 rows plot, row to row distance 22.5 cm, and four replications) and then the superior lines were promoted to AVT (Advanced Varietal Trial, conducted zone wise in country, ∼24 locations, 6 m × 12 rows, row to row distance 22.5 cm, and four replications, data for Punjab locations have been given) and tested consecutively for 2 years before being identified for release. The complete procedure for testing and release of a variety in the Indian system is explained in Supplementary Figure 1. In order to shorten the variety release process, the coordinated program has created a special niche for direct entry of gene-pyramided lines into AVT which accelerates the release of resistant varieties. These special trials were named the ‘Marker-Assisted Backcross Breeding Near-Isogenic Lines (SPL-MABB/NIL) trials.

The data for yield and other traits from a coordinated trial (NIVT and AVT) were submitted to the Indian Institute of Wheat & Barley Research (IIWBR), Karnal, Haryana, India where it was analyzed and documented in ‘Crop Improvement reports’ are using standard statistical methods. The alpha–lattice experimental design was used and the mean values were compared using CD (critical difference) which is the same as LSD (least significant difference). One year of additional testing after identification of a genotype for release at the national level was completed at farmer fields in Punjab before recommending the variety for cultivation in the state.

## Results

The present study elucidates the step-wise work to introgress individual genes and then stack these genes for stripe rust and leaf rust resistance in wheat cultivar PBW343. The results are discussed individually with respect to the mobilization of leaf rust resistance to PBW343, the introgression of single stripe rust resistance genes in PBW343, the pyramiding of both leaf rust and stripe rust-resistant genes in PBW343, and the development and current status of PBW723 (*Unnat* PBW343), an improved version of PBW343 released at the national level.

### Mobilization of Leaf Rust Resistance to PBW343

The introgression of leaf rust resistance genes *Lr24* and *Lr28* was our first attempt to improve PBW343. This involved the development of near-isogenic lines individually for both genes in the background of PBW343 (Chhuneja et al., 2005). Leaf rust resistance gene *Lr24*, transferred from *Agropyron elongatum* to the long arm of wheat chromosome 3D through a spontaneous translocation, and *Lr28*, transferred from *Aegilops speltoides* to the long arm of wheat chromosome 4A, through induced homoeologous recombination, were still highly effective in the year 2020 against all the prevalent pathotypes of leaf rust in Punjab. The successful pyramiding of these genes in PBW343 showed a broad spectrum of leaf rust resistance and marked the second step in improving leaf rust resistance of the variety (Chhuneja et al., 2011). The BC_5_F_4_ PBW343 + *Lr24* + *Lr28* NILs had the potential to be used as a replacement for PBW343. However, when evaluated in preliminary yield trials, BW9250 had complete foliage protection from leaf rust owing to the presence of *Lr24* and *Lr28*, but showed a susceptible reaction to the stripe rust race pathogen 78S84 with virulence for *Yr27*. Similarly, BW9270 with introgression for *Lr57/Yr40* was completely resistant to leaf rust but showed a moderate resistant reaction for stripe rust. These lines had low yields as compared to check varieties on account of disease damage (Table 1).

**TABLE 1:** Yield performance and rust score for selected lines with different gene introgressions at PAU, Ludhiana.

S. No.	Genotype	Yield (q/ha)	PBW 621	CD	Stripe rust	Leaf rust	Year of testing
1	BW9250 (PBW343 + *Lr24* + *Lr28*)	41.2	45.5**	2.3 2.7	60S	0	2008–2009 2009–2010
2	BW9270 (PBW343 + *Lr57/Yr40*)	47.8 41.4	40.2	1.8 2.3	20S 40S	0 0	2009–2010 2010–2011
3	BWL3274 (PBW343 + *Lr24* + *Lr28* + *Yr5*)	59.6	64.3***	2.1	0	0	2011–2012
4	BWL3275 (PBW343 + *Lr24* + *Lr28* + *Yr5*)	63.5			0	0	2011–2012
6	PBW343	35.7			60S	40S	2011–2012

** Check PBW343. *** Check as PBW621.

### Mobilization of Improved Stripe Rust Resistance in PBW343

#### Introgression of Gene *Lr37/Yr17/Sr28* to PBW343: Development of PBW665

The gene *Lr37/Yr17/Sr28* was transferred into PBW343 and the homozygous high yielding resistant lines from the cross were identified. The segregated generations were screened under stripe rust epiphytotics for the selection of resistant progenies. From this set, the best selected disease resistant and agronomically elite line PBW665 was evaluated at multiple locations in Punjab during 2009–2010 and was nominated to NIVT during 2010–2011, based on its performance and resistance to stripe rust. Further, PBW665 was promoted to AVT during the year 2011–2012, and its performance was evaluated across 24 diverse locations in the NWPZ. The data for trials conducted by PAU at five locations in Punjab are given in Table 2. PBW665 performed significantly better at Ludhiana (69.2 qt/ha, cd/LSD = 5) and Gurdaspur (54.3 qtl/ha), while it was statistically on par with PBW343 at other locations. PBW665 showed statistically significant yield superiority and resistance to stripe rust over its recurrent parent PBW343. Another wheat genotype, DPW621-50 (KAUZ//ALT84/AOS/3/MILAN/KAUZ/4/HUITES) identified from a CIMMYT nursery and internationally known as ‘KACHU,’ also carrying the 2NS (*Lr37/Yr17/Sr38*) gene complex, was tested in the same years along with PBW665. PAU and IIWBR both identified the same line and entered it into the coordinated trials as PBW621 and DBW50, respectively. Being the same genotype, it was tested as a joint entry under the name DPW621-50. PBW665 was superior to PBW343, but could not out yield DPW621-50 (66.3 qt/ha) in Punjab. Further, the stripe rust score for PBW665 soared to 100S at the Dhaulakuan location (Table 2), thus rendering it susceptible to stripe rust. However, a genotype carrying a similar 2NS gene complex but with a different genetic background, DPW621-50, was resistant with a highest score of 10S at the same location. The leaf rust resistance gene *Lr37* was effective toward prevalent leaf rust pathotypes.

**TABLE 2:** Yield performance and rust score of PBW665 in AVT at different locations across Punjab during 2011–2012.

Yield performance (quintals/hectare) at five locations in Punjab										Rust (Leaf rust: mixture of races 77-5 and 104-2) and (Stripe rust: mixture of races 78S84 and 46S119) reaction at two locations in Punjab			

Locations ⇒	Bathinda	Gurdaspur	Kapurthala	Ludhiana	Rauni	State mean (Punjab)	Zonal mean (NWPZ)	Ludhiana		Gurdaspur		Dhaulakaun	
Genotypes ⇓								Br	Yr	Br	Yr	Br	Yr
PBW665 *(*PBW343 + *Lr37/Yr17/Sr28)*	54.9	54.3	57.5	69.2	67.4	62	51.5	0	0	0	10S	0	100S
PBW343	66.3	30	55	60	66.6	55.6	50.5	10S	10S	0	30S	0	60S
DBW17	65	51.4	54.9	54.7	64.9	58.2	54.7	10S	10S	0	30S	0	0
DPW621-50	67.1	62.3	59.7	71.7	70.7	66.3	55.4	0	0	0	5S	0	10S
G.M	62.1	56.2	55.5	62.1	62.3	0.843	0.322						
C.D.(10%)	6	3.7	2.9	5	5.1	2.3	0.9						

*G.M, general mean of trial; C.D., critical difference/least significant difference; NWPZ, North Western Plain Zone; Br, brown rust; Yr, yellow rust.*

#### Introgression of Genes *Lr76/Yr70* in PBW343: Development of PBW683

A newly identified and characterized gene from *Aegilops umbellulata* (now designated as *Yr70/Lr76*, Kuraparthy et al., 2007; Bansal et al., 2017, 2020), allelic to gene *Lr57/Yr40*, transferred from *Aegilops geniculate* into hexaploid wheat WL 711 was targeted for introgression in PBW343. Both being alien segments from non-progenitor species, they were introgressed in wheat on the short arm of chromosome 5D, a recombination hot spot. A large set of introgression lines containing *Yr70/Lr76* in the PBW343 background were generated following phenotypic selection for plant type and MAS for genes of interest. Advance breeding lines PBW683, PBW693, and PBW701 were selected from a larger introgression set to be tested in NIVT during 2011-12 at 17 locations across the NWPZ and North Eastern Plain zone (NEPZ). PBW683 (57.8 qtl/ha) was significantly superior to its recurrent parent PBW343 and was numerically superior but statistically on par with the best check in the NWPZ (DBW17, 55.8 qt/ha) with rust resistance (Table 3) ensuring its promotion to testing under AVT during 2012–2013 at 24 locations across the NWPZ including five locations in Punjab (Table 3). PBW343 was not used as a check in the national trials as new varieties (HD2967 and WH1105) were introduced for comparison in trials. However, during the very next year of testing, the PBW683 yield (47.3 qtl/ha) was lowest at all locations across Punjab whereas WH1105 (MILAN/S87230/Babax) had the highest yield with a Punjab average of 60.4 qtl/ha. The low yield could be attributed to stripe rust susceptibility as PBW683 reported a score of 40S (Table 3) against the prevalent races in Punjab. Even the stripe rust score for DPW621-50 and DBW17 had enhanced to 20S and 60S, respectively. The new variety WH1105 showed a 5S reaction for stripe rust. The entry PBW683 (PBW343 + *Yr70/Lr76)* got rejected at this stage owing to stripe rust susceptibility.

**TABLE 3:** Yield performance and rust score of PBW683 in AVT at different locations across Punjab during 2012–2013.

Yield performance (quintals/hectare) at five locations in Punjab								Rust (Leaf rust: mixture of races 77-5 and 104-2) and (Stripe rust: mixture of races 78S84 and 46S119) reaction at two locations in Punjab			

Locations ⇒	Ludhiana	Bathinda	Rauni	Gurdaspur	Kapurthala	State mean (Punjab)	Zonal Mean (NWPZ)				
								Ludhiana		Gurdaspur	
									
Genotypes ⇓								Br	Yr	Br	Yr
PBW683 *(*PBW343 + *Lr76/Yr70)*	46	49.1	49.7	41.9	49.6	47.3	50.6	0	40S	0	40S
DPW621 –50	64.8	52.5	57.1	53.1	52	55.9	53	0	20S	0	20S
HD2967	72.7	53.8	56.5	57.1	54.7	59	53.3	0	10S	0	20S
DBW17	62.1	55.7	55.2	34.7	52.8	52.1	50.6	0	60S	0	60S
WH1105	75.6	50.3	57.5	58.1	60.6	60.4	53.9	0	10S	0	5S
GM	62.6	52.3	54.8	48.4	55.5						
CD	6.2	4.7	3.3	2.6	2	2	1				

*G.M, general mean of trial; C.D., critical difference/least significant difference; NWPZ, North Western Plain Zone; Br, brown rust; Yr, yellow rust.*

#### Introgression of Gene *Yr5*: Development of BWL3274 and BWL3275

Gene *Yr5*, incorporated individually in BW9250 (*PBW343* + *Lr24* + *Lr28*), is the leaf rust-resistant version of PBW343. Gene *Yr5* is known to be transferred from the *Triticum aestivum* sub species *spelta* and located on the long arm of chromosome 2B. It has shown resistance to all prevalent pathotypes of stripe rust in India through 2020. BW9250 was crossed with Avocet6^∗^/*Yr5*, and agronomically desirable plants homozygous for all the three genes were selected and bulked for testing in station trials at PAU. The high yielding, disease-resistant lines BWL3274 and BWL3275 were tested at three different locations (Bathinda, Ludhiana, and Gurdaspur) in the Punjab state (data not given). These lines showed yield on par with the check variety as well as complete resistance (immune reaction) to stripe rust (*Yr5*) and leaf rust (*Lr24* + *Lr28*) as compared to PBW343, which was reported to have an 80S and 60S score for stripe and brown rust, respectively, at all three locations. However, these trials included other breeding lines that had a better yield than these two (BWL3282, released in 2015 as PBW725) and therefore were held back from being nominated to NIVT.

#### Pyramiding of Genes *Yr10* + *Yr15* + *Lr24* + *Lr28*: Development of PBW698 and PBW703

The genes *Yr10* and *Yr15* were individually transferred to the PBW343 + *Lr24* + *Lr28* (BW9250) background using marker-assisted selection. The agronomically elite derivatives carrying genes *Yr10* + *Lr24* + *Lr28* were inter-mated with PBW343 + *Yr15* + *Lr24* + *Lr28* derivatives. Figure 1 describes the step-wise transfer and MAS for this convergence. A large number of *F*_1_ seeds were produced and backcrossed to BW9250 to generate sufficient BC_1_F_1_ plants for each cross. Two simultaneous backcrossing program were followed to generate BC_1_F_1_’s of both the crosses which were subjected to phenotypic selection for plant type (optimum flowering time, plant height, tillers per plant, spikelets per spike, grain appearance, etc.) and artificial stripe rust infection to ensure the presence of *Yr10* and *Yr15* genes individually. The stripe rust-resistant plants were selected and were confirmed to have *Lr24, Lr28*, and *Yr10* as well as for *Lr24, Lr28*, and *Yr15* genes in respective sets using molecular markers. The selected BC_1_F_1_ plants were subsequently backcrossed with BW9250 to generate BC_2_F_1_ plants. This generation was again subjected to stringent phenotypic as well as molecular marker selection to confirm both the leaf rust resistance and stripe rust resistance genes in the respective crosses. Agronomically elite plants with two leaf rust and one stripe rust resistance (*Yr10* and *Yr15* in respective BC_2_F_1_s) genes in a homozygous state were intercrossed to pyramid the four genes. A total of 4755 seeds were planted and stripe rust free phenotypically good plants were first identified and was subjected to molecular marker-based screening. Artificial epiphytotic for stripe rust created at the seedling stage identified the susceptible plants indicating the absence of any *Yr* gene. Such plants (∼297) were uprooted at the seedling stage. The DNA was extracted from the remaining 4458 plants. The gene *Lr24* was confirmed using a linked molecular marker and the plants positive for *Lr24* were screened for the presence of *Lr28*. The parrot-green ear color of the plants which turn into reddish brown glumes nearing maturity is a morphological marker for the *Yr10* gene (Metzger and Silbaugh, 1970). This was used to identify plants with the *Yr10* gene in addition to the *Lr24* and *Lr28* genes. Now, the shortlisted plants with genes *Yr10*, *Lr24*, and *Lr28* individually or together were tested for the presence of gene *Yr15* using linked markers. The presence of gene *Yr10* was also confirmed using molecular markers. Plants with dark green foliage and ears but resistant to stripe rust were confirmed using molecular markers for only the presence of gene *Yr15*. The cross resulted in the generation of lines with different gene combinations *Yr10 (130), Yr15 (121), Yr10* + *Yr15 (218), Yr10* + *Yr15* + *Lr24 (212), Yr10* + *Yr15* + *Lr28 (225), Yr10* + *Lr24* + *Lr28 (210), Yr15* + *Lr24* + *Lr28 (137), and Yr10* + *Yr15* + *Lr24* + *Lr28* (71). All the lines carrying *Yr10*, *Yr15*, and *Yr10* + *Yr15* with *Lr24 and Lr28* were tested in station trials at PAU for yield testing. Lines varied in their performance and a selected set of lines were tested at multiple locations. Superior entries were finally nominated to national trials. PBW702 (PBW343 + *Yr15* + *Lr24* + *Lr28*), PBW698, and PBW703 (*PBW343* + *Yr10* + *Yr15* + *Lr24* + *Lr28)* were entered into NIVT 2012-13 for national testing, while PBW712 (*PBW343* + *Yr10* + *Yr15* + *Lr24* + *Lr28)* was nominated into the coordinated program a year later. These lines were resistant to all prevalent pathotypes of both leaf and stripe rust pathogens in the NWPZ. Both lines PBW698 and PBW703 were resistant to the 78S84 and 46S119 pathotypes of stripe rust and 77-5 and 104-4 pathotypes of leaf rust at the seedling stage and adult plant stage. The recipient parent PBW343 was susceptible to all races of stripe and leaf rust at the seedling stage and adult plant stage. Based upon the yield advantage and disease resistance to both leaf and stripe rusts at different locations, PBW698 and PBW703 both got promoted to the Advance Varietal Trial (AVT-I) in their respective planting conditions. PBW698 was on par in yield in Punjab (59.2 qtl/ha) and the NWPZ (55.3 qtl/ha) with the checks WH1105 yielding 59.9 qtl in Punjab and 56.3 qtl/ha at the NWPZ level. DBW88 and HD3086, the new wheat varieties for NWPZ were identified at national level and were used as check varieties in trials (Table 4). Since PBW698 did not out yield the best check statistically and numerically, it was dropped from the next stage of trials. PBW703 was statistically on par with the best check in Punjab. However, it yielded significantly lower than the best check (HD3059) in the cumulative data of the zone and therefore could not be promoted for further testing and identification as a varietal candidate. The yields of PBW698 and PBW703 could be attributed to a high level of resistance to rust, especially stripe rust. The rust score for PBW698 was 20MS with an average coefficient of infection (ACI) of 3.8, whereas PBW343 totally succumbed to stripe rust with an 80S score and an ACI of 31.6. The rust score for PBW703 was 20MS with an ACI of 2.3 which is less than PBW343 (80S with an ACI of 31.6).

**TABLE 4:** Yield performance (quintals/hectare) of PBW698 in AVT timely sown and PBW703 in AVT late sown at different locations across Punjab and zonal average during 2013–2014.

Testing under timely sown conditions							Testing under late sown conditions						
Genotypes	Ludhiana	Bathinda	Gurdaspur	Kapurthala	State mean (Punjab)	Zonal mean) (NWPZ)	Genotypes	Ludhiana	Kapurthala	Gurdaspur	Bathinda	State mean (Punjab)	Zonal mean) (NWPZ)
PBW698 *(PBW343* + *Yr10* + *Yr15* + *Lr24* + *Lr28)*	58.6	65.8	53.2	59.2	59.2	55.3	PBW703 (PBW343 + *Yr10* + *Yr15* + *Lr24* + *Lr28)*	55.5	43.2	49	47.2	48.7	46.9
HD2967	54	54.4	37.4	57.1	50.7	51.5	WH1021	29.3	31.1	25.5	42.9	32.2	40.3
DPW621-	60.8	57.2	46.2	56.5	55.2	54.7	HD3059	53.4	44.6	47.6	50	48.9	48.6
WH1105	62.7	63.1	54.5	59.4	59.9	56.3	PBW590	28.8	33.8	22.3	50.7	33.9	40
DBW88	65.8	67.2	46.6	59.8	59.9	53.9	WH1124	49.4	42.3	46.4	46.1	46	46
HD3086	59.7	68.6	49	60.6	59.5	55.9	DBW90	53.3	40.1	45	48.6	46.8	46.9
G.M	58	60	46.7	57.1			G.M	46.4	38	40.1	48.5		
C.D (10%)	6.3	7.3	4.8	6.5	3.1	1.2	C.D (10%)	4.2	4.5	4.3	5.1	2.2	0.8

*G.M, general mean of trial; C.D., critical difference/least significant difference; NWPZ, North Western Plain Zone.*

#### Pyramiding of Genes *Lr37/Yr17/Sr38* and *Lr76/Yr70*: Development of PBW723 (*Unnat* PBW343)

A modified MABB strategy was used to pyramid the genes *Lr37/Yr17/Sr38* and *Yr70/Lr76* into PBW343 (PBW665/PBW683) with five genes, two genes each for leaf and stripe rust and one gene for stem rust resistance. The gene segments were first incorporated individually in the PBW343 background and then a converging cross was attempted to stack all the genes together. During development of the individual lines, three backcrosses were made and therefore, no backcross was made in the pyramiding step. A large set of lines was generated and BWL3345, a line developed using MABB strategy and selected after local yield evaluation was nominated into SPL-MABB/NIL in 2013-14 as PBW723. PBW722, another line selected after local and state trials from a larger set of material generated in an attempt to pyramid *Yr15* with *Lr57/Yr40*, was also nominated into SPL-MABB/NIL in 2013-14 along with PBW723. The trial was conducted at six locations in the country namely Haryana (Karnal), Punjab (Ludhiana), Himachal Pradesh (Dhaulakuan), Uttarakhand (Pantnagar), Jammu & Kashmir (Jammu), and New Delhi in four replications in a randomized block design. The data for Punjab are given along with the zonal average in Table 5 (Tiwari V. et al., 2014). PBW723 was promoted to AVT in the second year (2014-15), whereas PBW722 was dropped from AVT in the first year. PBW723 was further tested at the national level for an additional year (2015–2016) before its identification as the varietal candidate to be released. During 2013–2014, PBW723 (48.7 qtl/ha), significantly out-yielded the recipient variety PBW343 (35.6 qtl/ha) with a 36.79% yield increase and was on par with check variety HD2967 (49.4 qtl/ha). However, it was statistically inferior to the best check WH1105 with a 7.23 percent decrease in yield. PBW722 was on par with PBW723 and numerically superior as well, however, owing to some accidental seed mixture, it was rejected while PBW723 was promoted. During 2014–2015, PBW723 (48.5 qtl/ha) out-yielded HD2967 (44.2 qtl/ha) with 9.72% yield superiority and PBW343 (41.7 qtl/ha). It was statistically on par with DPW621-50 (46.5 qtl/ha) and statistically lower than WH1105. Initially the trial was meant to test for 2 years to accelerate the varietal identification and release process, but with rust appearing on WH1105 and a lesser number of sites of testing in this newly formed trial, an additional testing year was recommended for the PBW723 entry. PBW723 maintained its yield level during the 3 years of testing. It possessed the lowest ACI for stripe rust under natural and artificial conditions and a very high level of resistance to leaf rust over the years 2013–2014 to 2015–2016 (Table 6). PBW723 was the only entry in the trial exhibiting seedling resistance to all the four isolates (two of stripe rust and two of leaf rust) while PBW343 was susceptible to all and other checks showed susceptibility to two or three of the isolates (Table 7). Also, the adult plant response (APR) against individual pathotypes was resistant against all the predominant pathotypes of yellow and leaf rust for PBW723. Based upon the performance at the national level from the crop seasons 2013–2014 to 2015–2016, it was identified to be released as a new variety for cultivation under timely sown irrigated conditions in the NWPZ. In Punjab, an additional test in a farmer’s field for PBW723 was done on 87 locations across 20 districts of Punjab (Table 8). The overall mean yield of PBW723 (54.3 qt/ha) gave a 7.7 percent enhancement over PBW343 and a 0.4 percent over HD3086. In trials conducted by the State Department of Agriculture, a farmer, ‘Jagroop Singh’ from the village Dhudhike, District Moga, Punjab reported a yield of 72.0 quintals per hectare for PBW723. The variety was released for cultivation in 2017 and was given another name by PAU for Punjab: *Unnat* PBW343, where the regional word, *Unnat* is locally used in Hindi/Punjabi/Urdu and means improved. The perception was that such nomenclature would relate the new version to the original variety and its practices in the eyes of the farmers to raise a good crop.

**TABLE 5:** Yield performance (quintals/hectare) of PBW723 in SPL-MABB trials at different locations.

	2013–2014			2014–2015			2015–2016		
	State mean (Punjab)	Zonal mean) (NWPZ)	% Increase	State mean (Punjab)	Zonal mean) (NWPZ)	% Increase	State mean (Punjab)	Zonal mean) (NWPZ)	% Increase
PBW722 *(PBW343* + *Yr15* + *Lr57/Yr40)*	44.4	48.8		–	–		–	–	
PBW723 *(PBW343 Lr37/Yr17/Sr38* + *Yr70/Lr76)*	49.3	48.7		63.3	48.5		59.5	49.8	-
PBW343	23.8	35.6	+36.79	48.3	41.7	+16.31	54.4	42.0	+18.57
DPW621-50	42.6	47.7	+2.1	62.6	46.5	+4.3	55.7	49.8	0
HD2967	45.5	49.4	–1.42	50.4	44.2	+9.72	54.7	46.9	+6.18
WH1105	52.6	52.5	–7.23	60.9	50.9	–4.71	62.0	51.8	–3.86
GM	42.4			57.1	46.3	–	57.2	47.9	–
C.D(10%)	3.9	2.1		6.5	2.2	–	8.2	1.5	–

*G.M, general mean of trial; C.D., critical difference (least significant difference); NWPZ, North Western Plain Zone.*

**TABLE 6:** Reaction of PBW723 to stripe and leaf rust under natural and artificial conditions at seedling and adult plant stage from 2013–2014 to 2015–2016.

Disease/condition	Year	PBW723		PBW343	
		HS	ACI	HS	ACI
**Stripe rust**					
Natural field conditions (no inoculations or epiphytotic conditions created)	2013–2014	20S	5.0	80S	31.6
	2014–2015	20S	4.4	80S	55.0
	2015–2016	10S	5.0	80S	54.0
Artificial conditions (creation of epiphytotic conditions using a mixture of prevalent pathotypes 78S84 and 46S119)	2013–2014	10S	3.3	100S	58.9
	2014–2015	20S	6.4	100S	63.0
	2015–2016	20S	8.4	100S	69.0
**Brown rust**					
Natural field conditions (no inoculations or epiphytotic conditions created)	2013–2014	0	–	0	–
	2014–2015	0	–	0	–
	2015–2016	–	–	–	–
Artificial conditions (creation of epiphytotic conditions using a mixture of prevalent pathotypes 77-5 and 104-2)	2013-2014	0	–	40S	8.1
	2014–2015	10S	1.3	100S	21.0
	2015–2016	TS	0.1	40S	10.5

*– Indicates comparable data not available. HS, highest score; ACI, average coefficient of infection.*

**TABLE 7:** Rust reaction of PBW723 at seedling stage against prevalent stripe and brown rust pathotypes.

Year	Rust	Pathotype	PBW723	PBW343
2014–2015	Stripe rust	78S84	R	S
		46S119	R	S
		110S119	–	–
		238S119	–	-
	Brown rust	77-5	R	MS
		77-9	R	R
		104-2	R	S
2015-2016	Stripe rust	78S84	R	S
		46S119	R	S
		110S119	MS	S
		238S119	S	S
	Brown rust	77-5	R	MS
		77-9	R	R
		104-2	R	MS

**TABLE 8:** Evaluation summary of PBW723 in farmers’ fields during year 2016–2017.

Trial	Number of total trials conducted	PBW723 (PBW343 *Lr37/Yr17/Sr38* + *Yr70/Lr76*)	Commercial checks		
			PBW343	HD3086	PBW725
PAU extension services	67	54.5	51.1	54.2	53.3
External agency (State Department of Agriculture)	20	53.5	48.0	53.6	53.7
Mean	87	54.3	50.4	54.1	53.4
% increase over check		–	+7.7	+0.4	+1.7

## Discussion

Among biotic stresses, the fungal pathogens causing foliar rusts in wheat continually evolve and overcome host plant resistance. Wheat breeders therefore, face the endless task of continually developing new wheat varieties combining enhanced yield and disease resistance. In this research article, we discuss in detail the efforts and attempts made to develop a rust-resistant version of a popular wheat variety, PBW343. The paper sheds light on attempts made, failures, and insights on the development and release of wheat variety *Unnat* PBW343.

### Breakdown of Genes *Yr27* and *Yr17* Against New Pathotype and Current Status of Effective Genes

Stripe rust incidence in Punjab over the last one and a half decade has demonstrated a progressive shortening of the life span of wheat varieties. PBW343 succumbing to the stripe rust pathotype 78S84 became a breeding nightmare for wheat breeders and pathologists. The large area under its cultivation and the absence of any equivalent yielding substitute made it vital to temporarily resolve the situation using chemical control. Emergent recommendations for the use of fungicides were made and popularized. The new stripe rust race 110S119 emerged and progressed aggressively turning DBW17, PBW 550, and other varieties susceptible before gaining popularity as cultivars. Meanwhile, the entire breeding conduit with stripe rust resistance based on the *Yr27* gene collapsed (Bhardwaj et al., 2016). Nevertheless, the genes with resistance for the newly emerging races were available in rust differential stocks in the Avocet background and included genes *Yr5, Yr10, and Yr15*. In addition, the resistant gene complex *Yr17/Lr37/Sr38* was available through the international CIMMYT nursery. Also, the new stripe rust and leaf rust-resistant genes *(Yr40/Lr57 and Yr70/Lr76)* had been identified from wild species of wheat at PAU and were under the process of mapping and tagging at that time. While on the other hand, PBW343 was being grown under fungicide protection, and was still popular among farmers. In the present scenario, gene *Yr17* is no longer effective in Punjab and the situation is similar for *Yr70* which individually gave a 40S reaction for stripe rust. However, these genes tend to provide resistance when stacked together. Starting from a breakdown of gene *Yr27*, the pathogen has been evolving continually as shown by the prevalence of the stripe rust races 78S84, 46S119 to 110S119, and currently 238S119. In 2005-06, PBW343 was the highest yielding cultivar so naturally PBW343 was used as a base for the introgression of known genes for rust resistance. Major wheat breeding efforts were targeted to develop the NILs at PAU with all individual or pyramided stripe rust resistance genes in the PBW343 background. *Yr10*, originally from the wheat cultivar Moro possesses seedling resistance and is located on the short arm of chromosome 1B (Metzger and Silbaugh, 1970). The gene *Yr15*, originated from *Triticum dicoccoides* and its linked molecular markers are available (Peng et al., 2000) to facilitate MAS for both these genes. PBW703 displayed almost complete foliage resistance to rusts (stripe and leaf) and high yield, however it was not superior in yield potential when compared with the newer national check varieties and hence, could not be released as a variety. PBW703 has been registered (Registration number-INGR 15052) as a stock with the National Bureau of Plant Genetic Resources (NBPGR), Govt. of India. It is the nodal organization of the country for the management of plant genetic resources which acquires and organizes the indigenous as well as exotic germplasm collection to ensure sustainable use and exchange of plant genetic resources for crop improvement. PAU also has a simple ‘on request’ germplasm exchange policy under which PBW703 has already been shared and is being extensively used by wheat breeders across the country as the source of four rust-resistant genes.

The current scenario in Punjab has rendered most of the stripe rust genes (*Yr9, Yr17, Yr27, Yr31, Yr40, Yr51, and Yr70*) ineffective individually and calls for gene stacks. The genes currently effective in India are *Yr5*, *Yr10, Yr15, Yr47, Yr57, and Yr63* (Sohu, 2019, unpublished internal annual reports). The cultivars released in the NWPZ for cultivation namely, PBW502, DBW17, PBW550, PBW621, HD2967, and WH1105 all showed their first incidence of stripe rust within 3 years of release (Tiwari et al., 2012, 2013, 2015, 2016; Tiwari V. et al., 2014). However, HD3086 released in 2015 lost its avirulence during 2019–2020 (Singh et al., 2020). In the post-PBW343 era, multiple cycles of pathogen racial evolution have occurred, thereby reducing the average resistant time span of varieties. In such a situation, the time spanning the initiation of mobilizing a resistance gene to the release of a resistant cultivar usually is too long for a gene to remain resistant. This phenomenon has been very clear in the case of PBW665 and PBW680 in our study. Responding to this emergent situation, special effort by a coordinated program through the creation of a niche for the direct entry of gene pyramided lines into AVT in the first year (SPL-MABB/NIL trials) in 2013 proved very useful. PBW723 was entered into this special trial for national level testing.

### Efficacy of MAS for Gene Pyramiding in Wheat

Single gene introgressions are usually performed using backcrossing to transfer the targeted gene into the recurrent parent. While developing the individual gene lines, phenotypic selection and disease epiphytotic conditions were given more value than the application of markers. Plants selected in field were confirmed for the presence of the gene of interest, while no markers were applied to the initial segregated generations. The use of genes in combination, irrespective of whether they are major or minor genes, has been suggested as the best method for the genetic control of rusts (Roelfs, 1988). Gene pyramiding is difficult when using conventional breeding methods, however, the availability of molecular markers closely linked with the target genes makes the identification of plants with two or more genes possible (Chhuneja et al., 2011). Pyramiding of major genes using MAS in agronomically suitable cultivars is being extensively pursued (Samsampour et al., 2009; Revathi et al., 2010; Vinod et al., 2010; Bhawar et al., 2011; Chhuneja et al., 2011; Tiwari S. et al., 2014; Khan et al., 2017) to prevent the breakdown of resistance in major crop breeding programs all over the country. A marker-assisted background selection strategy proposed by Randhawa et al. (2009) reports maximum recurrent parent (RP) genome recovery in the MABS derived line WA8059. Genotypic comparison between WA8059 and WA8046 (the BC4F7 line WA8046 was developed using the same two parents using backcross breeding with phenotypic selection without MABS) reported 97% of the recurrent parent recovery as compared to 82% in WA8046. Three genes for blast resistance have been introgressed using MABS in an aromatic rice cultivar (Khan et al., 2018) with 92% RP genome recovery. MABS makes it possible to recover a high level of the RP genome and helps in the improvement of a specific trait in otherwise elite cultivars within a short time span. Moreover, this method almost nullifies the linkage drag in case the donor for the trait of interest is genetically distant or agronomically inferior. But applying MABS in the breeding program requires excessive and high throughput molecular marker work. The chances of recovering the RP will however decrease with the increase in the number of genes, especially if they are on different chromosomes.

Extensive MABS was not used in the development of PBW723, rather the percentage of RPG was assessed in a set of selected introgression lines using molecular markers. The selections during the development of PBW723 were based upon agronomically desirable plant type and PBW343 look-alike plants. Such selected plants were subjected to marker-assisted foreground selection for genes of interest and further, the phenotypically selected plants with stacked genes were tested for grain traits before generation advance. The foreground markers were again confirmed in the next generation and the bulks were yield tested. The bulk that became PBW723 was the best one and had superior yielding among its other sibs and therefore, was chosen to assess the background recovery with regards to PBW343. This modification in the MABS strategy (Randhawa et al., 2009) saved time and the financial resources required for generating marker data points by testing more sibs that were not to be used for varietal testing. The polymorphic markers with either parent or RP recovery based on them was 85.80 and 85.34 percent, however the overall recovery was 81.57 percent (Tables 9, 10). The development of PBW723 was initiated using the well-established, yield-tested advanced breeding lines PBW665 and PBW683. The component lines had undergone three backcrosses with the recurrent parent that empirically implies around 93.125 percent similarity with PBW343. However, since the marker-assisted background recovery was never attempted in those lines and only phenotypic similarity was considered in segregating generations during their development, the RP genome recovery is lower than those theoretically targeted or expected using the MABS strategy in the case of PBW723. No additional backcrosses with PBW343 were made while developing PBW723, rather the single genes introgressed in PBW343 were converged to combine the genes. The introgressions for the genes in this case were on two different chromosomes (2AS and 5DS) and were alien in nature, which further reduced the background recovery. Nevertheless, PBW723 is phenotypically close to PBW343 in terms of plant type, flowering time, maturity days, spike shape, spike size, and grain shape, etc. Later, while initiating its nucleus seed program, single ear purification was re-evaluated and 11 sibs were identified and tested along with PBW723. One of the sibs (PBW723#13) outperformed the original PBW723 and had a longer ear with a higher grain number. However, another sib (PBW 723#8) was more phenotypically similar to PBW343 and it was chosen as the final source for its nucleus seed production. Phenotypic similarity with RP, resistance level, and yield were given more value over marker-assisted background recovery. This modification and balance made the varietal development economically tenable and easy for adoption even for the low budget breeding programs. The MABS approach can be of use in cases where field evaluation is not possible or the trait of interest cannot be easily assessed phenotypically. However, for the case of breeding wheat for rust resistance, the modified MABS protocol is equally promising and more economical. More specifically, if the donor parents and recurrent parent are agronomically well-adapted and have superior high phenotypic similarity among each other (as in the case of PBW723), background recovery using molecular markers can be minimized for selected progeny rather than applying molecular background selection to segregated generations.

**TABLE 9:** Brief summary of molecular markers used for background recovery in PBW723.

Chromosome #	A	B	D	Total	
1	24	28	20	72	
2	31	37	27	95	
3	28	35	22	85	
4	26	26	19	71	
5	28	31	26	85	
6	24	31	20	75	
7	36	27	29	92	
	197	215	163	575	

**Class of SSRs used**					

**Wmc**	**barc**	**gwm**		**cfd**	**cfa**

182	146	104		123	20

**TABLE 10:** Percent background of PBW343 recovered in PBW 723.

Total markers applied	Polymorphic between PBW343 and WL711 + *Lr57/Yr40*	Polymorphic between PBW343 and Thatcher + *Lr37/Yr17*	*Polymorphic between PBW343, WL711 + Lr57/Yr40 and Thatcher + *Lr37/Yr17*	Recipient (PBW343) markers in PBW723	Percent recovery of PBW343 background in PBW723
575	185	–	–	158	85.40
575	–	191	–	163	85.34
575	–	–	255**	208	81.57

*The number of molecular markers polymorphic between recipient and either donor or between all three parental lines. **Excluding the markers associated with target genes.

### Status of PBW723 Against Leaf and Stripe Rust

The response of individual genes and their contribution to enhanced rust resistance toward different prevalent pathotypes of yellow and brown rust post pyramiding was studied and the results are presented in Table 11. Stripe rust resistance gene *Yr17* showed high levels (1550–2100) of AUDPC (area under disease progress curve) in response to the stripe rust pathotypes 46S119 and 110S119. The virulence/avirulence of these races for the genes under consideration is given in Supplementary Table 3. The response of *Yr70* toward the same races was lower than that of *Yr17* (AUDPC levels 525–1150) as it provides partial resistance to stripe rust. Cultivar PBW343 had the highest AUDPC (2400) against the three pathotypes in both the years. The AUDPC score for stripe rust was greater in 2018–2019 compared to 2017–2018. The test cultivar PBW723 had the lowest AUDPC scores over both years. When tested against a mixture of pathogen races (46S119 and 110S119), the response was lower, however individually *Yr70* had three times the AUDPC (625) compared to PBW723 (200). Also, the cultivar PBW621 possessing *Yr17* had a six times higher AUDPC (1250) compared to PBW723. PBW723 with *Yr17* and *Yr70* showed enhanced resistance whereas lines with only one of the genes were susceptible. We hypothesize that *Yr17* and *Yr70* interact epistatically to enhance stripe rust resistance in PBW723.

**TABLE 11:** Role of defeated genes in contributing resistance to stripe rust.

Genotype	Pedigree	AUDPC*					
		46S119		110S119		Mixture of races	
		2017–2018	2018–2019	2017–2018	2018–2019	2017–2018	2018–2019
PBW682	PBW343/Tc + *Lr37/Yr17*//3^∗^PBW343	2000	2000	1550	2000	2100	2000
PBW665	PBW343/Tc + *Lr37/Yr17*//3^∗^PBW343	2000	2000	1550	2000	2100	1800
PBW683	WH890-*Ae*. *umb*3732 amph/CS (S)//WL711 NN/3/3^∗^PBW343	1050	950	625	625	525	1150
PBW621	Kauz//Altar84/AOS/3/Milan/auz/4/Huites	1450	1250	1700	1250	1450	1300
PBW723	PBW343/Tc + *Lr37/Yr17*//3^∗^PBW 343/4/WH 890-*Ae*. *umb* 3732 amph/CS (S)//WL711 NN/3/3^∗^PBW 343	250	150	325	200	525	850
PBW343	ND/VG9144//KAL/BB/3/YACO“S”/4/VEE#5	2400	2400	2400	2400	2400	2400

*AUDPC, ‘Area under disease progress curve’, calculated as suggested by Wilcoxson et al. (1975).

### Concept of Ghost Resistance

Minor genes or the defeated genes in PBW343 also added somewhat toward resistance when combined with other genes. This kind of additive effect of minor genes has also been shown to provide resistance in segregating generations of two susceptible cultivars (Singh et al., 2017). They reported that in the two crosses involving stripe rust susceptible parents (PBW 621 × PBW343 and HD 2967 × PBW343) the resistant segregants possessed two genes, one contributed by PBW621 or HD2967 (depending on the cross) and the other is hypothesized to be from the most susceptible cultivar, PBW343. The susceptible parent PBW343 contributing toward a resistant phenotype is referred to as ghost resistance, in which a defeated gene contributes toward resistance when combined with other genes. Singh et al. (2017) reported that the ineffective stripe rust resistance gene (*Yr27)* present in PBW343 was able to contribute significantly when combined with minor genes from PBW621 as well as HD2967. Probably, the minor genes with additive effects conferred stripe rust resistance in highly resistant lines, as the widely reported additive nature of minor genes is known to confer stripe rust resistance (Parlevliet, 2002; Dutta et al., 2008). Singh et al. (2017) also hypothesized that when the resistance of an aggressive race breaks down in a variety and makes it completely susceptible, it seems to cause the breakdown of only a subset of diverse quantitatively determined resistance mechanisms. Susceptible lines have the potential to contribute some resistance when recombination provides an opportunity for a new assemblage of resistance components. This kind of resistance contributed by defeated major genes indicates the so-called ghost resistance hypothesis. The “defeated” or “ghost” resistance finds a mention in the literature (Nass et al., 1981; Li et al., 1999) and there are several compelling pieces of evidence which indicate that the allelic variants of major resistance genes account for a proportion of quantitative disease resistance in plants (Young, 1996). The pathology studies on PBW723 along with parent variety PBW343 at PAU demonstrated that the defeated individual genes when pyramided together can contribute to a high level of resistance based on AUDPC scores. The stripe rust resistance genes from both the segments had shown susceptibility in individual lines PBW665 and PBW683, however when stacked together, the set of pyramided lines (with genes *Lr37/Yr17/Sr38* and *Yr70/Lr76 in a PBW343 background)* were resistant to the stripe rust. PBW723 was the line selected from this set for further national level testing. Moreover, PBW723 was the only entry in the trial at the national level exhibiting seedling resistance to all the four isolates (two of stripe rust and two of leaf rust) while PBW343 was susceptible to all and other checks showed susceptibility to two or three of the isolates of the stripe rust pathogen. Based on these observations, we hypothesize that the stripe rust resistance genes *Yr17* and *Yr70* (the defeated genes individually) conferred resistance in PBW723.

### Status of PBW723 (Unnat PBW343) as a Cultivar

Post release, the variety PBW723 has made its way to farmers’ fields and is being grown in Punjab. It is used as a check variety in local trials at PAU and its average yield ranges from 55–60 qtls/ha, while some farmers reported yields as high as 75 qtls/ha. The acceptance and popularity of the variety among farmers can be assessed by its seed sale and demand (Supplementary Table 8). The seed of a variety after release is commercially available to farmers by PAU. More than 11,000 quintals of seeds have been produced in the last 2 years and seed sold during 2017–2018 equaled 11,393 quintals while in 2018–2019 it was 9345 quintals (Supplementary Table 8). Priced at a very nominal rate of ∼$33 per quintal, it gave a return of $375,969 and $308,385 to PAU in 2017–2018 and 2018–2019, respectively. PBW723 has held the third place at the National Breeder Seed Indent after HD2967 (10th year of cultivation) and HD3086 (7th year of cultivation), consecutively during the last 3 years. About nine percent of wheat cropped in Punjab is under PBW723 (survey of 1500 farmers, PAU reports, unpublished).

### Current Status of the Genes in PBW723

PBW723 carries five rust resistance genes at two loci: *Yr17/Lr37/Sr38* from *Aegilops ventricosa* on chromosome 2AL and *Yr70/Lr76* from *Aegilops umbellulata* on chromosome 5DS. The linked genes *Yr17/Lr37/Sr38* conferring resistance against stripe rust, leaf rust, and stem rust respectively, have been extensively used by wheat breeders in different parts of the world (Dyck and Lukow, 1988; McIntosh et al., 1995; Robert et al., 1999; Seah et al., 2000). This block of linked genes was initially introgressed in the winter wheat VPM1 from Triticum ventricosum (Maia, 1967) and is located on a 2NS/2AS translocation (Bariana and McIntosh, 1993; McIntosh et al., 1995). Stripe and leaf rust races with virulence to *Yr17* and *Lr37* have been identified in different countries but this gene cluster still provides resistance to a wide range of races in combination with other rust resistance genes (Helguera et al., 2003). *Yr17* provides partial resistance to prevalent stripe rust pathogens in India (Bhardwaj, 2011; Bhardwaj et al., 2016). Based on APR against individual pathotypes, PBW723 possessed resistance against all predominant pathotypes of yellow and brown rusts. PBW723 also has enhanced resistance to Karnal bunt (data not given) compared to recipient variety PBW343 as well as other check varieties (HD2967, DPW621-50, and WH1105). The 2NS chromosome segment (*Yr17/Lr37/Sr38)* has also been reported to be associated with significant reductions in head blast incidence in wheat under natural epidemic conditions in the field. But, not all cultivars and lines with 2NS showed resistance under controlled inoculations in the greenhouse (Cruz et al., 2016). The CIMMYT cultivar KACHU (carrying Milan, Kohli et al., 2011) possesses the 2NS translocation, and Milan-based resistant wheat cultivars released in South America appear to contain high levels of resistance to wheat head blast under field conditions (Kohli et al., 2011). This resistance is present in most of the germplasm in India through KACHU sourced from CIMMYT. Thus, PBW723 having this 2NS segment already gives a preemptive breeding product against the blast disease. In addition to effective rust and blast resistance, the 2NS/2AS translocation brings additional value to the wheat breeding program as it also carries resistance genes *Rkn3* and *Cre5* against root-knot nematodes (*Meloidogyne* spp., Williamson et al., 2013), and the cereal cyst nematode (*Heterodera avenae* Wollenweber, Jahier et al., 2001).

## Conclusion

The pipeline of our wheat breeding program is well fed by the germplasm developed using MAS for gene(s) of interest in diverse backgrounds and the modified MABS for reviving the promising varieties. The story focussing the journey of PBW343 to *Unnat* PBW343 has been through several failures and gains. Selection of genes to be pyramided and the selection of the appropriate background for specific genes has been a great learning experience. Genes *Yr10 and Lr28* probably were not the right choice in terms of genes for introgression in PBW343. The genes *Yr10 and Yr15* are both located on the 1B chromosome, so, they interfered with the 1B/1R translocation of PBW343 and it was observed that they put a ceiling on productivity traits. The gene *Lr28* is a large alien segment which compromises yield components. Therefore, the use of non 1B/1R lines in obtaining the maximum benefits of *Yr10* and the use of *Lr24* for ensuring leaf rust resistance is suggested. Once the selection of genes and background is appropriate, a critical balance between the population size, molecular marker input, and field testing needs to be established based on resources to effectively and economically incorporate MAS in the main breeding program. Contrary to the much prevalent notion that MAS may to some extent substitute the phenotypic characterization and selection done in conventional breeding (Huang et al., 2003), we learnt that the selections for plant type, resistance in field under relevant epidemics, and grain type selections after harvesting are important and may reduce the time and monetary inputs, making MAS/MABS feasible for small breeding groups. Molecular breeding and conventional breeding should be seamlessly combined together for the development of a resistant cultivar. Supplementary Figure 2 schematically summarizes the attempts involving the mobilization of single genes (for stripe and leaf rust), pyramiding events in the course of improving PBW343 for rust resistance, and the fate of this developed germplasm. All the lines are available at PAU on request. A similar kind of gene introgression work has already been completed in other genetic backgrounds like PBW550, DBW17, PBW621, HD2967, and HD3086, etc. and single gene introgression lines are being used for gene pyramiding. Also, the minor genes for resistance from CIMMYT material are being combined with major genes. The new genes from diverse sources like landraces, winter wheats, etc. are also being characterized and mobilized. The coordinated and continuous efforts of wheat breeders and pathologists will be required to win this unceasing battle against the rust fungi.

## Data Availability Statement

The original contributions presented in the study are included in the article/Supplementary Material, further inquiries can be directed to the corresponding author/s.

## Author Contributions

NB, AS, PS, VS, and PC conceived the theme of the study. PS, AS, SK, and GM completed the marker work and field evaluation. JK and RB completed the rust phenotyping. AS and PS drafted the manuscript. NB, PC, VS, and GS provided the overall guidance and edited the manuscript. TS generated and provided all post release data points regarding PBW723. All the authors read and approved the final version of the manuscript. AS, PS, and JK revised the manuscript during review.

## Conflict of Interest

The authors declare that the research was conducted in the absence of any commercial or financial relationships that could be construed as a potential conflict of interest.

## Supplementary Material

Supplementary Figure 1 Flow diagram showing the wheat varietal release procedure in India.

Supplementary Figure 2 Schematic representation depicting the summary of attempts involving the mobilization of stripe and leaf rust resistance in the course of improving PBW343 and the fate of this developed germplasm.
