# Supplementary figures and images for: Resurrection of Wheat Cultivar PBW343 Using Marker-Assisted Gene Pyramiding for Rust Resistance
Source: Front Plant Sci. 2021 Feb 11;12:570408. doi: 10.3389/fpls.2021.570408 (PMC7905314; doi:10.3389/fpls.2021.570408)

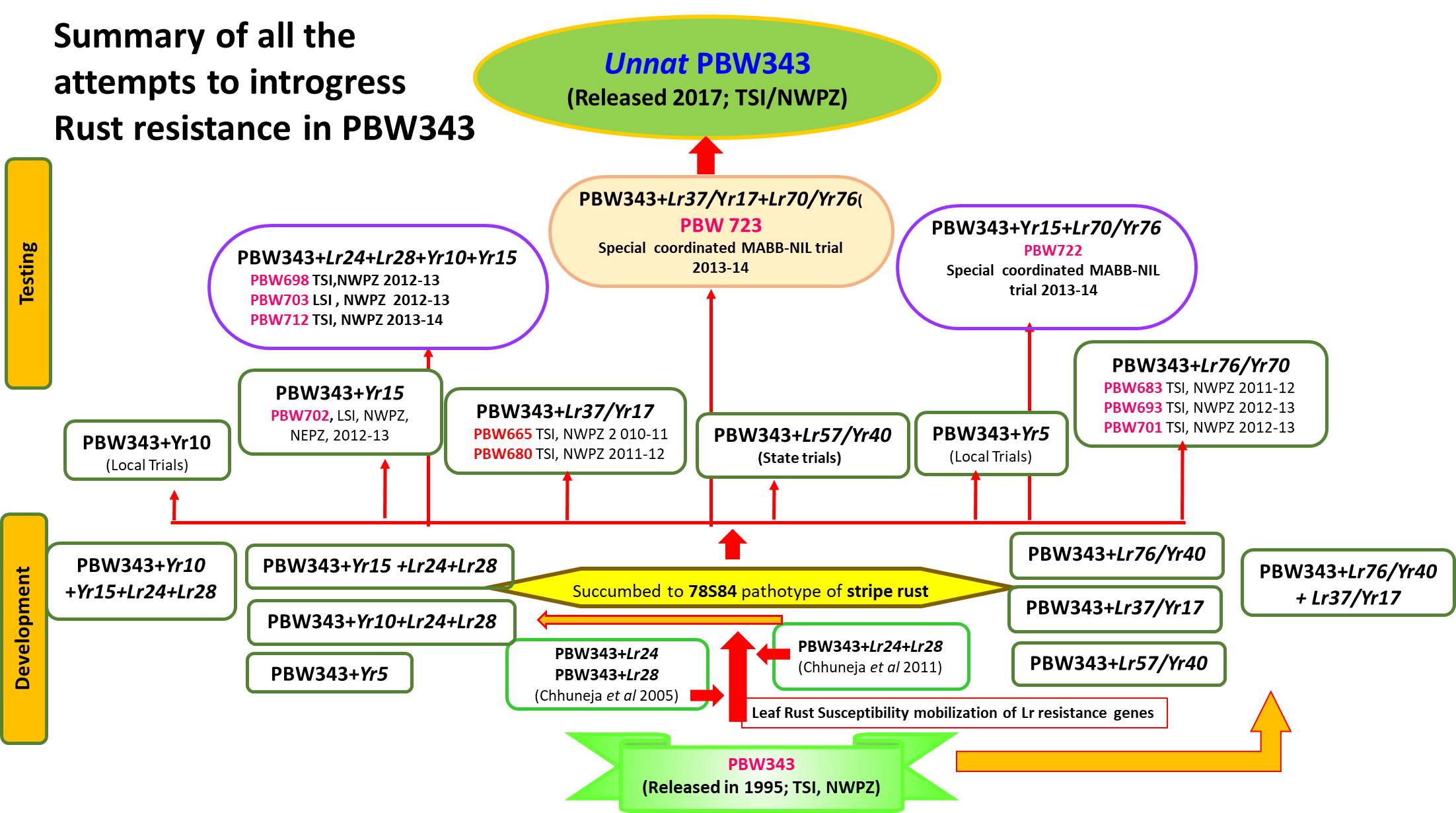

Supplement: Supplementary Figure 1 — Flow diagram showing the wheat varietal release procedure in India. [file Image_1.JPEG]

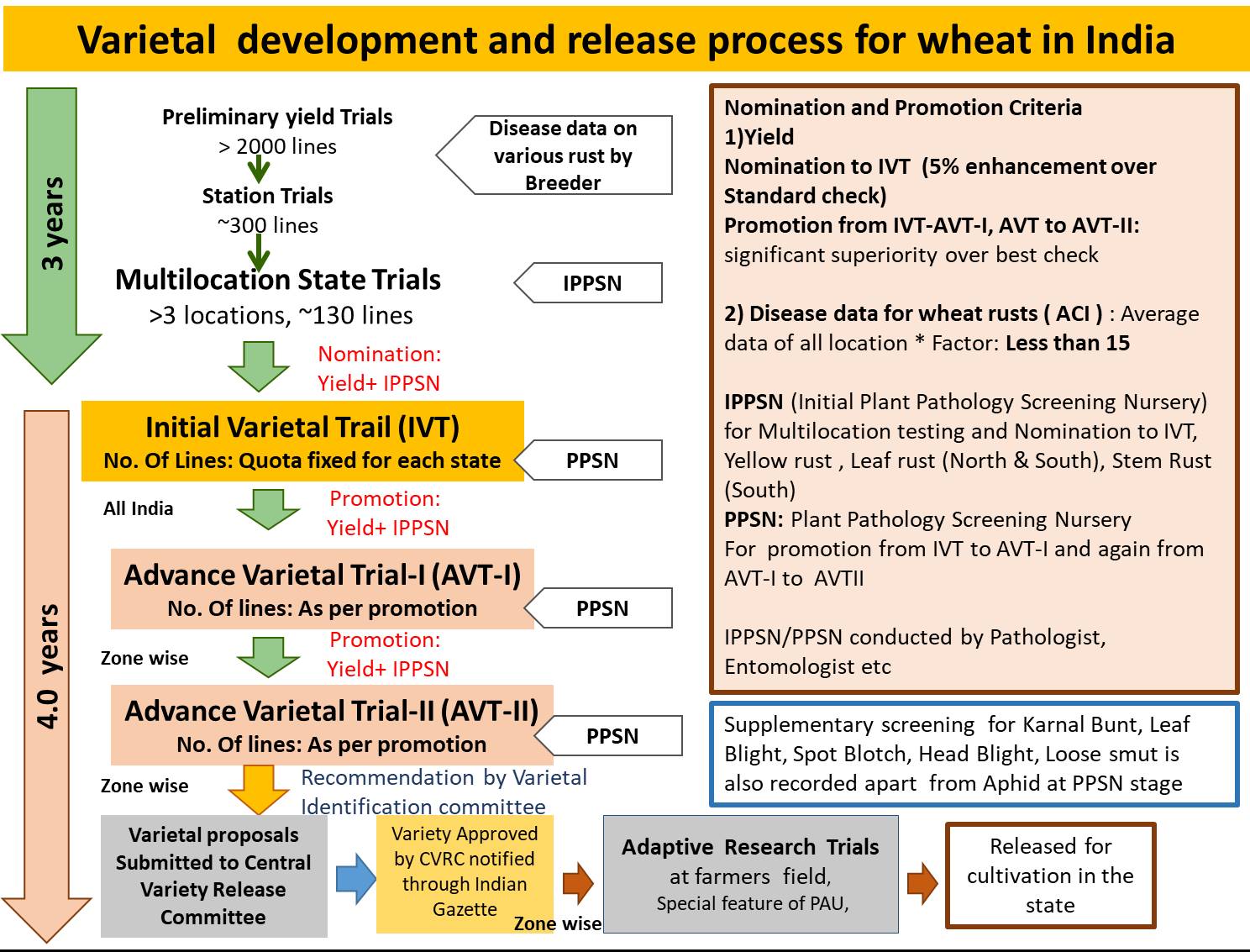

Supplement: Supplementary Figure 2 — Schematic representation depicting the summary of attempts involving the mobilization of stripe and leaf rust resistance in the course of improving PBW343 and the fate of this developed germplasm. [file Image_2.JPEG]
