# Supplementary material for: Resurrection of Wheat Cultivar PBW343 Using Marker-Assisted Gene Pyramiding for Rust Resistance
Source: Front Plant Sci. 2021 Feb 11;12:570408. doi: 10.3389/fpls.2021.570408 (PMC7905314; doi:10.3389/fpls.2021.570408)
Supplement: Supplementary file 3 [file Table_1.DOCX]

**Supplementary Table 1: Molecular markers used for incorporation of rust resistance genes into PBW343**

| **R genes used** | **R gene donor** | **Marker** | **Chromosome** | **Primer sequence** | **Reference** |
| --- | --- | --- | --- | --- | --- |
| ***Lr24*** | ***Agropyron elongatum*** | Xbarc71 | **3DL** | F:GCGCTTGTTCCTCACCTGCTCATA  R:GCGTATATTCTCTCGTCTTCTTGTTGGTT | **R.Mago et al, 2005** |
| ***Lr28*** | ***Aegilops speltoides*** | Xwmc313 | **4AL** | F:GCAGTCTAATTATCTGCTGGCG  R:GGGTCCTTGTCTACTCATGTCT | **S. Zheng et al, 2016** |
| ***Yr10*** | **Moro** | Xpsp3000 | **1BS** | F:GCAGACCTGTGTCATTGGTC R:GATATAGTGGCAGCAGGATACG | **Iqbal et al, 2016** |
| ***Yr15*** | ***Triticum turgidum var. dicoccoides*** | Xgwm 273 | **1BS** | F:ATTGGACGGACAGATGCTTT R:AGCAGTGAGGAAGGGGATC | **R. Rani et al, 2019** |
| ***Yr17/Lr37/Sr38*** | ***Aegilops ventricosa*** | Xgwm512 | **2AS** | F:AGCCACCATCAGCAAAAATT  R:GAACATGAGCAGTTTGGCAC | **M.S. Roder *et al,* 1998** |
| ***Yr70/Lr76*** | ***Aegilops umbellulata*** | Xgwm190 | **5DS** | F:GTGCTTGCTGAGCTATGAGTC  R:GTGCCACGTGGTACCTTTG | **J. Banyal *et al*, 2006** |
|  |  | *XTa5DS****-****2744983_kasp6* |  | **FAM:** GAAGGTGACCAAGTTCATGCTATAACCAGTCTGTCCACCCTTGAT  **VIC:** GAAGGTCGGAGTCAACGGATTATAACCAGTCTGTCCACCCTTGAC  **COM:** GAGTTATTATTCATCCGTCTGGG | **Bansal *et al*, 2017** |

**Supplementary Table 2: Modified Cobb scale (Peterson *et al* 1948)**

| **Category** | **Visible symptoms** |
| --- | --- |
| Immune | No visible infection on plant |
| R | Visible chlorosis or necrosis, no uredia are present |
| MR | Small uredia are present and surrounded by either chlorotic or necrotic areas. |
| M | Variable sized uredia are present; some with chlorosis, necrosis, or both |
| S | Large uredia are present, generally with little or no chlorosis and no necrosis |
| HS | Large uredia are present, generally with little or no chlorosis and no necrosis |

**Supplementary Table 3 Races/pathotypes of stripe and leaf rust used for screening the segregating materials over years and their virulence formula**

| **Race** | **Avirulence formula** | **Virulence formula** | **Prevalent in Years/comment** |
| --- | --- | --- | --- |
| **Leaf rust** | | | |
| 77-5 | *Lr9, Lr18, Lr19, Lr24, Lr25, Lr28, Lr29, Lr32, Lr39,Lr40, Lr40, Lr45* | *Lr1, Lr2a, Lr2c, Lr3, Lr10, Lr11, Lr12, Lr13, Lr14a, Lr14b, Lr14ab, Lr15, Lr16, Lr17, Lr20, Lr21a, Lr22b, Lr23, Lr26, Lr27+31, Lr30, Lr33,Lr34, Lr35, Lr36, Lr37, Lr38, Lr42, Lr43, Lr44, Lr48, Lr49, Lr76* | *Reported firstly in 1992 from Nilgiri. One of the most prevalent pathotypes till 2008-09* |
| 104-2 | *Lr9, Lr10, Lr13, Lr15, Lr19, Lr20, Lr24, Lr25, Lr28, Lr29, Lr32, Lr36, Lr39, Lr40, Lr43, Lr45* | *Lr1,Lr2a, Lr2b Lr3, Lr11, Lr12, Lr14a, Lr14b, Lr14ab Lr16, Lr17a, Lr18, Lr21, Lr22a, Lr22b, Lr23, Lr26, Lr30, Lr33, Lr34, Lr35, Lr37, Lr38, Lr42, Lr44, Lr48, Lr49, Lr76* | *Reported firstly in 1991 from Malan* |
| 77-9 | *Lr2a, Lr2b, Lr2c, Lr9, Lr19, Lr24, Lr25, Lr28, Lr32, Lr39, Lr45* | *Lr1, Lr3, Lr10, Lr11, Lr12, Lr13, Lr14a,Lr14b, Lr14ab, Lr15, Lr16, Lr17a, Lr17b, Lr18, Lr20, Lr21, Lr22a, Lr22b, Lr23, Lr26,Lr27* + *31, Lr30,Lr33, Lr34, Lr35, Lr36, Lr37, Lr38, Lr42, Lr44, Lr46, Lr48, Lr49* | *Reported firstly in 2008 from Belagavi and is now most prevalent pathotypes* |
| **Stripe rust** | | | |
| 78S84 | *Yr1, Yr 3b, Yr4b, Yr5, Yr10, Yr11, Yr14, Yr15, Yr17,, Yr18, Yr24/26, Yr28, Yr29, YrSD, Riebesel 147/51(Yr2,9,+)* | *Yr2, Yr3a, Yr4A, Yr6, Yr7, Yr8, Yr9, Yr12, Yr19, YrSk, YrSu, Yr3* | *First reported in 2001 from Batala, Punjab and was most prevalent till 2010-11* |
| 46S119 | *Yr1, Yr5, Yr10, ,Yr14, Yr15, Yr24, Yr26, Yr28, YrSp, Riebesel 147/51(Yr2,9,+)* | *Yr2, Yr3a, Yr3b, Yr4a,Yr4b, Yr6, Yr7,Yr8, Yr9, Yr11, Yr12,Yr 17, Yr19,Yr29, Yr31, Yrsk* | *First reported in 1996 from Gurdaspur district of Punjab and was most prevalent in Punjab till 2006-07* |
| 110S119 | *Yr1, Yr5, Yr10,Yr15, Yr24, Yr26, Yr28, YrSp, Riebesel 147/51(Yr2,9,+)* | *Yr2, Yr3a, Yr3b, Yr4a,Yr4b, Yr6, Yr7,Yr8, Yr9, Yr11, Yr12,Yr14, Yr 17, Yr18,Yr19,Yr29, Yr31, Yrsk, Yr70* | *Reported in 2014 from Ropar District of Punjab and is one of the prevalent races occurring in Punjab during the last 3-4 years* |
| 238S119 | *Yr1, Yr5, Yr10, Yr14,Yr15, Yr24, Yr26, Yr28, YrSp* | *Yr2, Yr3a, Yr3b, Yr4a,Yr4b, Yr6, Yr7,Yr8, Yr9, Yr11, Yr12, Yr 17, Yr18,Yr19,Yr29,Yr31,Yrsk, Riebesel 147/51(Yr2,9,+), Yr70* | *Reported in 2014 from Bilaspur from Himachal Pradesh and its prevalence is also increasing in Punjab every year from last 3-4 years* |

**Supplementary Table 4: Indian sets of differential genotypes for *Puccinia striiformis* f. sp. *tritici***

| **S. No.** | **Diﬀerential Genotypes** | | | | |
| --- | --- | --- | --- | --- | --- |
|  | **Set 0** | **Set A** | **Genes** | **Set B** | **Genes** |
| **1** | WH 147 | Chinese 166 | *Yr*1 | Hybrid 46 | *Yr*4 |
| **2** | Bilara | Lee | *Yr*7 | Heines VII | *Yr*2+ |
| **3** | WH 416 | Heines Kolben | *Yr*6, *Yr*2 | Compare | *Yr*8 |
| **4** | HD 2329 | Vilmorin 23 | *Yr*3 | *T. Spelta album* | *Yr*5 |
| **5** | HD 2667 | Moro | *Yr*10 | Tc*6/*Lr*26 | *Yr*9 |
| **6** | PBW 343 | Strubes Dickopf | *Yr*SD | Sonalika | *Yr*2+ |
| **7** | HS 240 | Suwon 92× Omar | *Yr*SU | Kalyansona (KS) | *Yr*2 |
| **8** | Anza | Riebesel 47/51 | *Yr*9+ |  |  |
| **9** | A-9-30-1 |  |  |  |  |

**Supplementary Table 5: List of SSR markers applied on parental and derivative lines during development of PBW723**

| **S.No** | **Marker** | **Chromosome** | **S.No.** | **Marker** | **Chromosome** |
| --- | --- | --- | --- | --- | --- |
| 1 | wmc826 | 1A, 4B, 7A | 47 | wmc339 | 1D |
| 2 | wmc51 | 1A,7B | 48 | cfd19 | 1D,5D,6D |
| 3 | wmc95 | 1A | 49 | cfd282 | 1D |
| 4 | wmc183 | 1A | 50 | barc99 | 1D |
| 5 | barc145 | 1A,3B,2D | 51 | cfd 27 | 1D |
| 6 | wmc 104 | ,6B | 52 | gwm 155 | 1D,2AL,2BS,3BL |
| 7 | gwm 136 | 1A | 53 | cfd 32 | 1D,3AL |
| 8 | gwm 164 | 1A | 54 | cfd19 | 1D |
| 9 | wmc 716 | 1A | 55 | wmc609 | 1D |
| 10 | cfa 2153 | 1A | 56 | barc149 | 1D |
| 11 | wmc336 | 1A,1D | 57 | barc169 | 1D |
| 12 | wmc24 | 1A | 58 | barc62 | 1D |
| 13 | cfd15 | 1A,1D | 59 | wmc489 | 1D,3A,4D,5A,7D |
| 14 | cfd22 | 1A,4B | 60 | gwm 232 | 1DL |
| 15 | barc17 | 1A | 61 | gwm 261 | 1DL |
| 16 | barc83 | 1A | 62 | gwm 642 | 1DL |
| 17 | wmc312 | 1A | 63 | wmc296 | 2A |
| 18 | Xgwm 135 | 1AL | 64 | wmc453 | 2A |
| 19 | cfa 2219 | 1AL | 65 | wmc702 | 2A,7D |
| 20 | gwm 497 | 1AL,2AS,3AL,3DL,5BL | 66 | wmc474 | 2A |
| 21 | gwm 33 | 1AS1BS,1DS | 67 | cfd168 | 2A,2D |
| 22 | wmc597 | 1B2B,4A,3B,6B,7A | 68 | cfd 86 | 2A,5D,5B |
| 23 | wmc269 | 1B,3A | 69 | gwm 356 | 2A,6A,7A |
| 24 | wmc498 | 1B,2B | 70 | gwm 614 | 2A,2DS |
| 25 | cfa 2129 | 1B,1A,1D | 71 | barc 220 | 2A,2B,2D,4A |
| 26 | cfd 20 | 1B,5B,7A | 72 | wmc63 | 2A |
| 27 | gwm 133 | 1B | 73 | wmc109 | 2A |
| 28 | cfd 65 | 1B,2A,5B | 74 | wmc261 | 2A,2B |
| 29 | gwm 268 | 1B,3A,4D,5B,6B,6D,7B | 75 | wmc632 | 2A |
| 30 | wmc 728 | 1B,4BL,6BS | 76 | wmc644 | 2A |
| 31 | wmc631 | 1B | 77 | wmc827 | 2A,3B,7D |
| 32 | wmc766 | 1B | 78 | cfd36 -1 | 2A,2D |
| 33 | wmc798 | 1B | 79 | barc124 | 2A,2B,2D |
| 34 | wmc830 | 1B | 80 | wmc149 | 2A,2B,5B |
| 35 | barc80 | 1B | 81 | wmc522 | 2A |
| 36 | barc128 | 1B,2B,3D,7D | 82 | cfd36 -2 | 2A,2D |
| 37 | barc8 | 1B | 83 | barc5 | 2A,6D,7D |
| 38 | barc60 | 1B,4B | 84 | barc76 | 2A,6B,7D |
| 39 | barc188 | 1B | 85 | gwm 311 | 2AL,2B,2D,6B |
| 40 | wmc694 | 1B | 86 | gwm 122 | 2AS |
| 41 | gwm 153 | 1BL | 87 | wmc477 | 2B |
| 42 | wmc 44 | 1BL | 88 | wmc770 | 2B |
| 43 | wmc 134 | 1BL | 89 | wmc441 | 2B |
| 44 | cfa 2147 | 1BL,1DL | 90 | barc18 | 2B |
| 45 | wmc 419 | 1BS | 91 | barc7 | 2B |
| 46 | cfd 2 | 1B,2D,2A,3D,4A,4D,5A,5B, | 92 | barc183 | 2B,6D |

Continued….

| **S.No.** | **Marker** | **Chromosome** | **S.No.** | **Marker** | **Chromosome** |
| --- | --- | --- | --- | --- | --- |
| 93 | gwm 388 | 2B | 138 | wmc153 | 3A |
| 94 | gwm 271 | 2B | 139 | wmc651 | 3A |
| 95 | wmc592 | 2B | 140 | wmc428 | 3A |
| 96 | wmc661 | 2B | 141 | barc67 | 3A |
| 97 | barc45 | 2B,3A | 142 | wmc 215 | 3AL,5DL |
| 98 | barc35 | 2B | 143 | cfa 2076 | 3AL |
| 99 | barc55 | 2B | 144 | cfd 79 | 3AS,3B |
| 100 | barc98 | 2B,4D | 145 | wmc679 | 3B,4B |
| 101 | barc13 | 2B | 146 | wmc43 | 3B |
| 102 | barc101 | 2B | 147 | wmc623 | 3B |
| 103 | barc159 | 2B,2D | 148 | cfd283 | 3B,4B,5D |
| 104 | barc200 | 2B | 149 | barc173 | 3B,6D |
| 105 | barc167 | 2B | 150 | barc84 | 3B |
| 106 | barc10 | 2B,4B | 151 | cfa 2226 | 3B,1A |
| 107 | gwm 257 | 2BS | 152 | wmc 754 | 3B |
| 108 | gwm 148 | 2BS,5A,5B,5D | 153 | barc77 | 3B |
| 109 | cfd16 | 2D | 154 | wmc808 | 3B |
| 110 | cfd 168 | 2D,2A | 155 | cfd4 | 3B,3D |
| 111 | cfd 175 | 2D,7D | 156 | cfd28 | 3B |
| 112 | cfd 73 | 2D | 157 | barc164 | 3B |
| 113 | gwm 349 | 2DL | 158 | wmc625 | 3B |
| 114 | cfd116 | 2D | 159 | barc73 | 3B |
| 115 | cfd233 | 2DL | 160 | wmc653 | 3B,7B |
| 116 | wmc601 | 2D | 161 | wmc693 | 3B |
| 117 | barc168 | 2D | 162 | barc68 | 3B,4B,3D |
| 118 | barc228 | 2D | 163 | barc87 | 3B,7D |
| 119 | wmc 181 | 2DL,2AL | 164 | wmc777 | 3B |
| 120 | wmc 817 | 2DL,2BL | 165 | barc75 | 3B |
| 121 | cfd 193 | 2DL,3AL,3DL,4DS,7AL,7DL | 166 | wmc 418 | 3BL,3DL |
| 122 | gwm 102 | 2DS | 167 | gwm 383 | 3BL,3D |
| 123 | wmc 470 | 2DS | 168 | gwm 154 | 3BL,5AL,7AS |
| 124 | wmc173 | 3A,4A | 169 | wmc 291 | 3BL |
| 125 | wmc169 | 3A | 170 | gwm 644 | 3BS,6BL,7BL |
| 126 | wmc505 | 3A,3B,3D | 171 | wmc656 | 3D |
| 127 | wmc532 | 3A | 172 | cfd141 | 3D |
| 128 | wmc559 | 3A | 173 | gwm 52 | 3D |
| 129 | wmc695 | 3A,3B | 174 | gwm 3 | 3D |
| 130 | barc69 | 3A | 175 | wmc492 -2 | 3D,5A |
| 131 | gwm 162 | 3A,4A | 176 | cfd152 | 3D |
| 132 | wmc 813 | 3A | 177 | cfd223 | 3D |
| 133 | barc 57 | 3A | 178 | barc52 | 3D |
| 134 | wmc96 | 3A,4A | 179 | barc125 | 3D |
| 135 | wmc388 -1 | 3A,5A,7A | 180 | wmc529 | 3D |
| 136 | wmc388 -2 | 3A,5A,7A | 181 | cfd 9 | 3DL |
| 137 | wmc640 | 3A,5B | 182 | cfd 35 | 3DS,1AL,2DL,5AL,5BS,5DL,7DS |

Continued….

| **S.No.** | **Marker** | **Chromosome** | **S.No.** | **Marker** | **Chromosome** |
| --- | --- | --- | --- | --- | --- |
| 183 | gdm 72 | 3DS | 228 | wmc713 | 5A |
| 184 | wmc15 | 4A | 229 | wmc805 | 5A |
| 185 | barc70 | 4A,7A,7D | 230 | barc 56 | 5A,5BL |
| 186 | gwm 565 | 4A,5D | 231 | wmc705 | 5A |
| 187 | gwm 269 | 4A,5D | 232 | wmc752 | 5A |
| 188 | barc 190 | 4A,5B | 233 | wmc150 | 5A,6A |
| 189 | wmc491 | 4A,4B | 234 | barc197 | 5A |
| 190 | wmc48 | 4A,4B | 235 | barc180 | 5A,3B |
| 191 | wmc258 | 4A,5B | 236 | barc186 | 5A |
| 192 | wmc283 | 4A,7A | 237 | wmc654 | 5A |
| 193 | wmc680 | 4A | 238 | cfd39 | 5A,4B,4D |
| 194 | wmc757 | 4A | 239 | barc141 | 5A |
| 195 | wmc760 | 4A | 240 | wmc 110 | 5AL |
| 196 | cfa2256 | 4A,7A | 241 | wmc 577 | 5AL |
| 197 | cfd257 | 4A | 242 | gwm 291 | 5AL |
| 198 | barc170 | 4A | 243 | gwm 293 | 5AL,5B,5D,7B |
| 199 | barc206 | 4A,6A,3B | 244 | wmc 415 | 5AL |
| 200 | wmc718 | 4A | 245 | wmc 524 | 5AL |
| 201 | gwm 111 | 4AL,6A,7BL,7DS,2B,7B | 246 | wmc235 | 5B |
| 202 | wmc238 | 4B | 247 | wmc734 | 5B |
| 203 | wmc546 | 4B,7B | 248 | cfd5 | 5B |
| 204 | barc25 | 4B | 249 | barc140 | 5B |
| 205 | gwm 495 | 4B,4AL,4DS | 250 | barc89 | 5B |
| 206 | wmc511 | 4B | 251 | wmc 75 | 5B |
| 207 | wmc254 | 4B,6A | 252 | gwm 408 | 5B,5DS |
| 208 | wmc657 | 4B | 253 | wmc73 -2 | 5B |
| 209 | barc109 | 4B,5B | 254 | wmc289 -1 | 5B |
| 210 | barc163 | 4B | 255 | wmc289 -2 | 5B |
| 211 | barc20 | 4B | 256 | wmc405 | 5B,7A |
| 212 | gwm 251 | 4BL,4D | 257 | barc59 | 5B,2D |
| 213 | wmc 617 | 4BL | 258 | wmc73 -1 | 5B |
| 214 | Xgwm 149 | 4BS,4D | 259 | barc21 | 5B |
| 215 | wmc 47 | 4BS,5AS,5BS | 260 | barc142 | 5B |
| 216 | wmc 125 | 4BS | 261 | gwm 554 | 5BL,2AL,1BL,7A |
| 217 | wmc399 | 4D | 262 | gwm 159 | 5BS |
| 218 | cfd23 | 4D | 263 | wmc357 | 5D |
| 219 | cfd40 | 4D | 264 | wmc636 | 5D |
| 220 | wmc331 | 4D | 265 | cfd26 | 5D |
| 221 | wmc720 | 4D | 266 | cfd29 | 5D |
| 222 | wmc825 | 4D | 267 | cfd156 | 5D |
| 223 | wmc 622 | 4DL | 268 | cfd 18 | 5D |
| 224 | barc 225 | 4DL | 269 | cfd 189 | 5D |
| 225 | cfd 84 | 4DL | 270 | cfd3 | 5D |
| 226 | gwm 608 | 4DS,1BS,1DL,6BL,2DL | 271 | cfd37 | 5D,6D |
| 227 | wmc475 | 5A,7B | 272 | cfd10 | 5D |

Continued…

| **S.No.** | **Marker** | **Chromosome** | **S.No.** | **Marker** | **Chromosome** |
| --- | --- | --- | --- | --- | --- |
| 273 | cfd183 | 5D | 319 | cfd 76 | 6BS |
| 274 | barc143 | 5D | 320 | wmc753 | 6D |
| 275 | barc144 | 5D | 321 | wmc822 | 6D |
| 276 | Lr57 | 5D | 322 | cfd42 | 6D |
| 277 | cfd266 -1 | 5D,7D | 323 | cfd219 | 6D |
| 278 | cfd266 -2 | 5D,7D | 324 | barc196 | 6D |
| 279 | gwm 182 | 5DL | 325 | cfd287 | 6D |
| 280 | cfd 8 | 5DL | 326 | barc54 | 6D |
| 281 | wmc553 | 6A | 327 | barc96 | 6D |
| 282 | wmc580 | 6A | 328 | barc204 | 6D |
| 283 | barc146 | 6A,6B | 329 | wmc 749 | 6DS |
| 284 | wmc 672 | 6A | 330 | wmc65 | 7A |
| 285 | cfd 30 | 6A,1A,4A,6D | 331 | wmc497 | 7A |
| 286 | barc37 | 6A | 332 | wmc593 | 7A |
| 287 | wmc145 | 6A | 333 | wmc633 | 7A |
| 288 | wmc243 -2 | 6A,2B | 334 | wmc809 | 7A |
| 289 | wmc256 -1 | 6A | 335 | cfa2049 | 7A |
| 290 | cfd190 | 6A,6D | 336 | wmc17 | 7A |
| 291 | wmc807 | 6A | 337 | cfd242 | 7A |
| 292 | wmc201 | 6A | 338 | wmc 479 | 7A |
| 293 | wmc243 -1 | 6A,2B | 339 | wmc 9 | 7A,1A |
| 294 | wmc243 -3 | 6A,2B | 340 | wmc 83 | 7A |
| 295 | wmc256 -2 | 6A | 341 | wmc596 | 7A |
| 296 | wmc417 | 6A,6B | 342 | wmc603 | 7A |
| 297 | gwm 169 | 6AL | 343 | wmc607 | 7A |
| 298 | wmc 684 | 6AL | 344 | wmc646 | 7A,7D |
| 299 | wmc748 | 6B | 345 | cfa2040 | 7A,7B |
| 300 | wmc786 | 6B | 346 | barc127 | 7A,6B |
| 301 | wmc152 | 6B | 347 | barc154 | 7A,7D |
| 302 | cfd1 | 6B,6D | 348 | wmc 790 | 7AL |
| 303 | wmc397 | 6B | 349 | cfa 2019 | 7AL |
| 304 | wmc398 -1 | 6B | 350 | cfd 2040 | 7AL,7BL,7DL |
| 305 | wmc398 -2 | 6B | 351 | gwm 60 | 7AS,7B |
| 306 | wmc486 | 6B | 352 | wmc218 | 7B |
| 307 | wmc539 | 6B | 353 | barc94 | 7B |
| 308 | cfd13 -2 | 6B,6D | 354 | barc123 | 7B |
| 309 | barc134 | 6B | 355 | barc85 | 7B |
| 310 | barc178 | 6B | 356 | barc176 | 7B |
| 311 | cfd13 -1 | 6B,6D | 357 | wmc 10 | 7B |
| 312 | wmc756 | 6B | 358 | wmc 70 | 7B |
| 313 | wmc105 | 6B | 359 | gwm 302 | 7B |
| 314 | wmc473 | 6B | 360 | barc32 | 7B |
| 315 | barc24 | 6B | 361 | barc72 | 7B |
| 316 | wmc 179 | 6BL,6AL,7AS,2BS | 362 | barc182 | 7B |
| 317 | gwm 193 | 6BS | 363 | wmc335 | 7B |
| 318 | cfd 13 | 6BS,6AS,7AS | 364 | wmc76 | 7B |

Continued..

| **S.No.** | **Marker** | **Chromosome** |
| --- | --- | --- |
| 365 | barc95 | 7B |
| 366 | wmc 396 | 7BL |
| 367 | wmc 723 | 7BL |
| 368 | gwm 537 | 7BS |
| 369 | wmc463 | 7D |
| 370 | cfd14 | 7D |
| 371 | barc111 | 7D |
| 372 | barc121 | 7D |
| 373 | wmc 14 | 7D |
| 374 | wmc797 | 7D |
| 375 | cfd21 | 7D |
| 376 | cfd31 | 7D |
| 377 | cfd175 | 7D |
| 378 | cfd41 | 7D |
| 379 | barc184 | 7D |
| 380 | wmc506 | 7D |
| 381 | cfd 25 | 7DL |

**Supplementary Table 6: List of 27 markers out of 381 applied showing introgression of Lr70*/Yr76* in PBW723**

in

| **S.No.** | **Marker** | **Chromosome location** |
| --- | --- | --- |
| 1 | barc188 | 1B |
| 2 | wmc 134 | 1BL |
| 3 | cfa 2147 | 1BL,1dl |
| 4 | wmc 419 | 1BS |
| 5 | barc167 | 2B |
| 6 | wmc601 | 2D |
| 7 | barc168 | 2D |
| 8 | barc228 | 2D |
| 9 | cfa 2076 | 3AL |
| 10 | wmc625 | 3B |
| 11 | barc73 | 3B |
| 12 | gwm 111 | 4AL,6A,7BL,7DS,2B,7B |
| 13 | wmc657 | 4B |
| 14 | barc109 | 4B,5B |
| 15 | wmc331 | 4D |
| 16 | wmc720 | 4D |
| 17 | cfd 84 | 4DL |
| 18 | barc186 | 5A |
| **19** | **Lr57 marker** | **5D** |
| **20** | **cfd266 -1** | **5D,7D** |
| **21** | **cfd266 -2** | **5D,7D** |
| 22 | wmc807 | 6A |
| 23 | wmc201 | 6A |
| 24 | cfd13 -1 | 6B,6D |
| 25 | gwm 537 | 7BS |
| 26 | cfd41 | 7D |
| 27 | barc184 | 7D |
|  |  |  |

*Markers associated with target gene introgression are given in bold face*

**Supplementary Table 7: List of 27 markers out of 381 applied showing introgression of *Lr37/Yr17* in PBW723**

| **S.No.** | **Marker** | **Chromosome** |
| --- | --- | --- |
| 1 | wmc312 | 1A |
| 2 | wmc 728 | 1B,4BL,6BS |
| 3 | wmc694 | 1B |
| 4 | wmc489 | 1D,3A,4D,5A,7D |
| **5** | **wmc149** | **2A,2B,5B** |
| **6** | **wmc522** | **2A** |
| **7** | **cfd36 -2** | **2A,2D** |
| **8** | **barc5** | **2A,6D,7D** |
| **9** | **barc76** | **2A,6B,7D** |
| 10 | barc10 | 2B |
| 11 | wmc653 | 3B |
| 12 | wmc693 | 3B |
| 13 | barc68 | 3B,4B,3D |
| 14 | wmc777 | 3B |
| 15 | wmc718 | 4A |
| 16 | barc163 | 4B |
| 17 | wmc825 | 4D |
| 18 | wmc654 | 5A |
| 19 | barc141 | 5A |
| 20 | barc21 | 5B |
| 21 | barc142 | 5B |
| 22 | wmc243 -1 | 6A,2B |
| 23 | wmc243 -3 | 6A,2B |
| 24 | wmc105 | 6B |
| 25 | wmc473 | 6B |
| 26 | barc24 | 6B |
| 27 | cfd 2040 | 7AL,7BL,7DL |
| 28 | wmc506 | 7D |

*Markers associated with target gene introgression are given in bold face*

**Supplementary Table 8: Seed production and sale of PBW723 by PAU, Ludhiana**

| **Production Year** | **Class of Seed** | **Production (q)** | **Sale as B/S** | **Sale as F/S** | **Sale as C/S** | **Sale as TL** | **Total Sale** | **National Breeder Seed indent** |
| --- | --- | --- | --- | --- | --- | --- | --- | --- |
| 2016-17 | B/S | 303.50 | 303.50 | - | - | - | 303.50 | = |
|  | F/S | - | - | - | - | - | - |  |
|  | C/S | - | - | - | - | - | - |  |
|  | Total | 303.50 | 303.50 |  |  |  | 303.50 |  |
|  |  |  |  |  |  |  |  |  |
| 2017-18 | B/S | 1590.70 | 1043.50 | - | - | 534.40 | 1577.90 | 46.8 |
|  | F/S | 4550.10 | - | 867.80 | 3679.80 | - | 4547.60 |  |
|  | C/S | 3882.80 | - | - | 3882.80 | - | 3882.80 |  |
|  | TL | 1384.90 | - | - | 1384.90 | - | 1384.90 |  |
|  | Total | 11408.50 | 1043.50 | 867.80 | 8947.50 | 534.40 | 11393.20 |  |
|  |  |  |  |  |  |  |  |  |
| 2018-19 | B/S | 1232.00 | 795.40 | - | - | 58.50 | 853.90 | 156.7 |
|  | F/S | 1692.00 | - | 164.40 | 847.40 | - | 1011.80 |  |
|  | C/S | 8151.04 | - | - | 7428.74 | - | 7428.74 |  |
|  | TL | 160.00 | - | - | - | 160.00 | 160.00 |  |
|  | Total | 11235.04 | 795.40 | 164.40 | 8276.14 | 218.50 | 9454.44 |  |

*B/S Breeder Seed, F/S Foundation Seed, C/S Certified Seed, TL Truthfully labeled*
